# Microtubule self-organisation during seed germination in *Arabidopsis*

**DOI:** 10.1186/s12915-020-00774-8

**Published:** 2020-04-30

**Authors:** Huifang Yan, Nicole Chaumont, Jean François Gilles, Susanne Bolte, Olivier Hamant, Christophe Bailly

**Affiliations:** 1 Laboratoire de Biologie du Développement, Sorbonne Université, CNRS, F-75005 Paris, France; 2 Key Laboratory of Pratacultural Science, Beijing Municipality, China Agricultural University, Beijing, 100193 China; 3 Grassland Agri-Husbandry Research Center, College of Grassland Science, Qingdao Agricultural University, Qingdao, 266109 China; 4 Imaging Core Facility, CNRS-FRE3631-Institut de Biologie Paris Seine, Sorbonne Université, F-75005 Paris, France; 5 Laboratoire de Reproduction et Développement des Plantes, Université de Lyon, ENS de Lyon, UCB Lyon 1, CNRS, INRA, F-69000 Lyon, France

**Keywords:** *Arabidopsis thaliana*, Cell elongation, Cortical microtubules, Dormancy, Germination, Seed

## Abstract

**Background:**

Upon water uptake and release of seed dormancy, embryonic plant cells expand, while being mechanically constrained by the seed coat. Cortical microtubules (CMTs) are key players of cell elongation in plants: their anisotropic orientation channels the axis of cell elongation through the guidance of oriented deposition of load-bearing cellulose microfibrils in the cell wall. Interestingly, CMTs align with tensile stress, and consistently, they reorient upon compressive stress in growing hypocotyls. How CMTs first organise in germinating embryos is unknown, and their relation with mechanical stress has not been investigated at such an early developing stage.

**Results:**

Here, we analysed CMT dynamics in dormant and non-dormant *Arabidopsis* seeds by microscopy of fluorescently tagged microtubule markers at different developmental time points and in response to abscisic acid and gibberellins. We found that CMTs first appear as very few thick bundles in dormant seeds. Consistently, analysis of available transcriptome and translatome datasets show that limiting amounts of tubulin and microtubule regulators initially hinder microtubule self-organisation. Seeds imbibed in the presence of gibberellic acid or abscisic acid displayed altered microtubule organisation and transcriptional regulation. Upon the release of dormancy, CMTs then self-organise into multiple parallel transverse arrays. Such behaviour matches the tensile stress patterns in such mechanically constrained embryos. This suggests that, as CMTs first self-organise, they also align with shape-derived tensile stress patterns.

**Conclusions:**

Our results provide a scenario in which dormancy release in the embryo triggers microtubule self-organisation and alignment with tensile stress prior to germination and anisotropic growth.

## Background

From the onset of their development, all organisms express a network of molecular regulators which behaviour is constrained by the physical properties of their local environment and by the forces that cells generate [1]. Typically, in higher plants, growth starts with the expansion of an embryo within a seed which is constrained by a stiff envelope [2]. Germination proceeds when dry seeds imbibe water and a growing radicle protrudes through the seed coat. Germination does not require cell division but is driven by elongation of cells of the embryonic axis [3, 4]. Water uptake, however, may not be sufficient to trigger germination. In particular, seed dormancy blocks their germination upon imbibition [5]. Dormancy is a transient phenomenon, dormant seeds being able to germinate only in a narrow range of environmental conditions. For example, dormant *Arabidopsis thaliana* seeds are usually unable to germinate above 20 °C in darkness [6], but cold stratification releases dormancy within a few days. The mechanism of seed dormancy is far from being understood. It is under the antagonist crosstalk of two plant hormones, abscisic acid (ABA) and gibberellins (GAs). ABA maintains dormancy whereas GAs stimulate seed germination [5].

Osmolytes and reserves make dry seeds highly hyperosmotic, meaning that cell walls and plasma membrane are likely experiencing reduced tensile stress and tension, respectively. Data on fixed tissues also suggest that microtubules are often absent in dry seeds (e.g. in dry *Jatropha curcas* seeds [7]). Seeds are also one of the rare plant tissues where β-tubulin is even not detected by western blots in certain species (e.g. in dry tomato seeds [8]). Upon water entry, cells rapidly switch to a new osmotic status that pressurises the cells and increases tensile stress in cell walls and tension in membranes. At this time, the embryo is mechanically constrained by the seed coat for at least a day or two, until the radicle protrudes through the envelopes. Turgor-dependent tensile stress in cell walls has been shown to affect the microtubular cytoskeleton: cortical microtubules (CMTs) tend to align with the direction of maximal tensile stress [9, 10]. This response has been observed in many plant tissues [11], including the seed coat [12], and is classically thought to promote the deposition of cellulose microfibrils, and thus to reinforce the cell wall to resist tensile stress in a feedback loop. A recent report shows how CMTs change their orientation in artificially compressed hypocotyls: using a custom-built automated confocal micro-extensometer, CMTs become transverse upon longitudinal hypocotyl compression [13]. Interestingly, CMTs also change their orientation in response to ABA and GAs. For instance, GAs induce the formation of transverse CMTs in pea epicotyls, whereas ABA suppresses this effect [14, 15]. This may involve key microtubule regulators, such as CLASP [16], SPIRAL1 and SPIRAL2 [17, 18], severing proteins such as KATANIN [19] and likely bundling proteins such as MAP65-1 and MAP65-2 [20].

The mechanical constraints applied by the seed coat on the imbibed embryo may therefore play a critical role in the dynamics of CMT organisation during seed germination. Surprisingly, this dynamics has never been described, and how the first CMT alignments appear during the transition from dry to germinating seed is not documented. In addition, whether regulation of seed germination by dormancy and hormonal balance may be related to CMT organisation and dynamics is not known. One indeed can wonder whether pressurisation of cells following water uptake has the same effect on cytoskeleton organisation in dormant and non-dormant seeds. The main objectives of this work were therefore to provide a description of CMT dynamics during the transition from dry to imbibed seeds and to determine whether it was regulated by dormancy. Transcriptome and translatome approaches were performed to determine whether microtubule self-organisation could be hindered by limiting amounts of tubulin and microtubule regulators. Studying CMT behaviour during germination was also a way to experimentally test predictions from [13] in a natural context. Based on our results, we propose a model in which dormancy release triggers CMT self-organisation and alignment with tensile stress during germination.

## Results

### Upon imbibition, CMTs first appear as few bundles in random orientations in hypocotyls from dormant seeds

As in the wild type, seeds from the *p35S::GFP-MBD* and *p35S::GFP-TUB6* marker lines used in this study displayed dormancy: they were unable to germinate at 25 °C in the darkness [6] (Additional file 1: Figure S1). Cold stratification progressively released seed dormancy of both lines (Additional file 1: Figure S1). Six hours of imbibition (HOI) was considered as an early time point where no elongation occurred. At 24 HOI, stratified seeds started to germinate (Additional file 1: Figure S1). In the following, we focus on the rootward region of the hypocotyl since most of the cell growth during *Arabidopsis* seed germination occurs in that region [3] (Additional file 2: Figure S2).

**Fig. 1:**
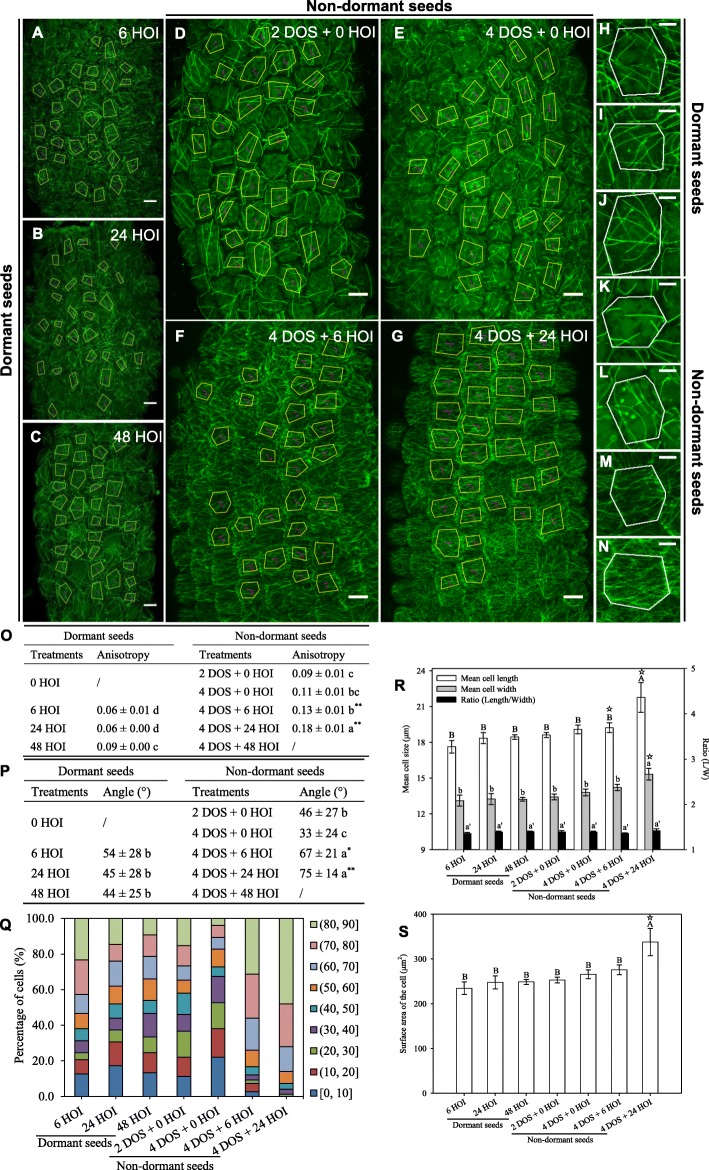
Microtubule organisation and hypocotyl cell shape in imbibed dormant and non-dormant *p35S::GFP-MBD* seeds. Microtubule average orientation (purple lines inside the cells) was obtained using FibrilTool in the regions of interest (cells excluding anticlinal walls) that are delineated with yellow lines (scale bars, 10 μm). Dormant seeds were imbibed at 25 °C in the dark for 6, 24 and 48 h (hours of imbibition (HOI)) or stratified for 2 and 4 days (days of stratification (DOS)) then imbibed for 6 and 24 h at 25 °C in the dark. Details of microtubule organisation are shown in individual cells of dormant embryos at 6, 24 and 48 HOI (**h**, **i**, **j**, respectively) and in cells of seeds after 2 DOS (**k**) and 4 DOS (**l**) followed by 6 (**m**) and 24 (**n**) HOI. Values of anisotropy (**o**) and angles (**p**) were calculated for the same samples. In (**o**, **p**) different letters indicated significant differences among all treatments (one-way ANOVA), and asterisks indicate significant difference between non-dormant seeds and dormant seeds at every the same time point (*t* test, *P < 0.05, **P < 0.01). **q** Angle distribution (expressed in %) among cells is shown for every sample. Changes in cell shape were evaluated by measuring cell length and width (**r**) and area (**s**), in which different letters indicate significant differences among all samples (one-way ANOVA), and stars indicate significant difference between non-dormant seeds and dormant seeds at every the same time point (*t* -test, ^☆^*P* < 0.05). Values of CMT array angles (**p**) are expressed as means ± s.d., and values of CMT array anisotropy (**o**) and cell shape (**r**, **s**) are expressed as means ± s.e. (5 biological replicates were analysed, and 30 cells from each replicate were used to calculate the anisotropy, angles and cell shape)

First, we analysed CMT behaviour using the *p35S::GFP-MBD* line in dormant seeds (Fig. 1). After 48 HOI of imbibition at 25 °C, no significant modification of hypocotyl cell morphology occurred in dormant seeds: cell length, width and surface did not change, consistent with the dormant status of these seeds (Fig. 1r, s). We quantified CMT orientation using the ImageJ macro FibrilTool [21]. Beyond average CMT orientation in each cell, this tool also provided a quantitative assessment of the anisotropy of the CMT arrays, where 0 means isotropic arrays and 1 means fully anisotropic arrays. Based on previous studies in hypocotyls, the measured anisotropy of CMT arrays rarely goes beyond 0.5 (see, e.g. [22]). At 6 HOI, a low number of microtubule bundles appeared, albeit without any clear orientation (Fig. 1a, h), in hypocotyl cells of dormant seeds. We found a low anisotropy of the CMT arrays (0.06, Fig. 1o) and rather isotropic CMT orientations (54 ± 28°, Fig. 1p, q). Longer durations of imbibition did not markedly modify these parameters: after 48 HOI, the anisotropy of the CMT arrays was equal to 0.09 and CMT average angle equal to 44 ± 25° (Fig. 1c, j, o–q).

**Fig. 2:**
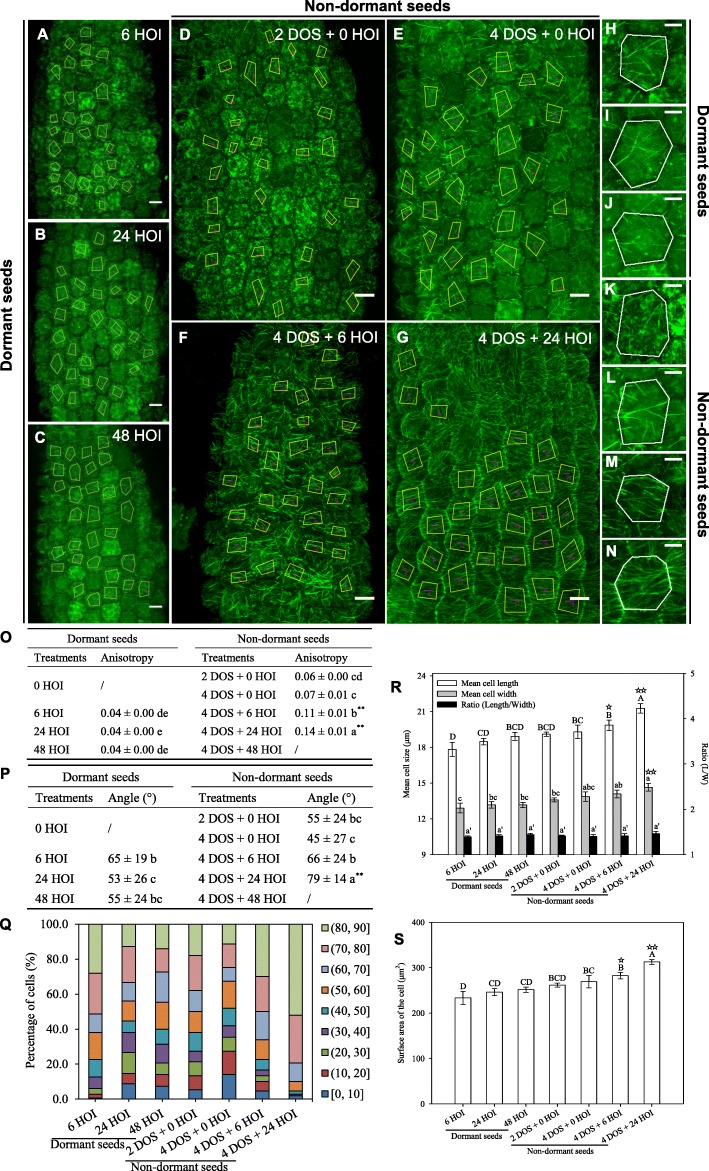
Microtubule organisation and hypocotyl cell shape in imbibed dormant and non-dormant *p35S::GFP-TUB6* seeds. *p35S::GFP-TUB6* seeds were used to confirm the findings shown in Fig. 1. Legends are similar to the ones in Fig. 1

The microtubule-binding domain of the *p35S::GFP-MBD* line increases the rate of microtubule bundling, potentially leading to artefacts. To check whether the results obtained above are independent of the construct used to image the microtubules, we next conducted the same analysis using the β-tubulin marker line *p35S::GFP-TUB6*. Overall, the analysis of CMT behaviour in the *p35S::GFP-TUB6* line confirmed the findings obtained with *p35S::GFP-MBD* line (Fig. 2). Upon imbibition of dormant seeds at 25 °C, *p35S::GFP-TUB6* CMTs formed very few scattered bundles, with no dominant orientation (Fig. 2a–c, h–j), as demonstrated by the relatively constant and low values of CMT array anisotropy and high standard deviation for CMT angle values (65 ± 19° at 6 HOI and 53 ± 26° at 24 HOI, Fig. 2o–q).

The presence of microtubule bundles could be explained by the sudden increase in mechanical stress, as water inflates the cells, as shown for instance when tissues are exposed to indentation (e.g. [23]) or after pharmacological wall weakening (e.g. [24]). Yet, at this stage, the low number of bundles suggests that the formation of CMTs is constrained by unknown factors.

### CMTs form close to transverse arrays in hypocotyls from germinating non-dormant seeds

Next, we performed the same analysis upon cold (4 °C) stratification for 2 and 4 days, to release dormancy, and after transfer in the dark at 25 °C (Figs. 1d–g). In the *p35S::GFP-MBD* background, the microtubule network features were not dramatically modified by 4 days of stratification (DOS): the anisotropy of the CMT array reached 0.11, and CMT orientations were still isotropic with a mean angle of 33 ± 24° (Fig. 1d, e, k, l, o–q), while cell morphology remained unchanged (Fig. 1r, s). However, as seeds progressed towards germination during imbibition at 25 °C, cell morphology changed with a significant increase in length, width and surface area (Fig. 1r, s). At 4 DOS and 24 HOI, the number of CMT arrays increased and the anisotropy of the CMT arrays reached 0.18, reflecting their increased parallel alignments. CMTs also displayed consistent orientations between neighbouring cells: the standard deviation of the CMT angle was down to 14° (Fig. 1o–q), and the mean angle of the CMT arrays was close to transverse, with a measured angle of 75° (Fig. 1p). This behaviour is consistent with predictions from [13] in artificially compressed hypocotyls. Similar conclusions were obtained when using the *p35S::GFP-TUB6* line (Fig. 2).

Altogether, these results demonstrate that germination is associated with an increase in the order of CMT arrays. In contrast, seed dormancy is associated with disorganised CMT arrays; water imbibition and cold treatment alone are not sufficient to trigger consistent CMT alignments.

### Germination is associated with stereotypical transcriptional and translational responses of microtubule-related genes

Upon water entry, the hypocotyl becomes pressurised, meaning that the cylindrical shape of the tissue may prescribe a maximum of tensile stress in the transverse direction for these cells. We wondered why CMTs would be insensitive to such directional cues in hypocotyls from dormant seeds. The alignment of CMT arrays in uniform orientations involves self-organisation processes: the final orientation of CMT arrays is the result of their interactions through (de)polymerisation, bundling and severing [25–27]. Following changes in tensile stress direction, CMTs reorient through such self-organising processes. We therefore reasoned that dormancy might hinder the expression of key microtubule regulators, thereby hampering the ability of microtubules to respond to stress.

**Fig. 3:**
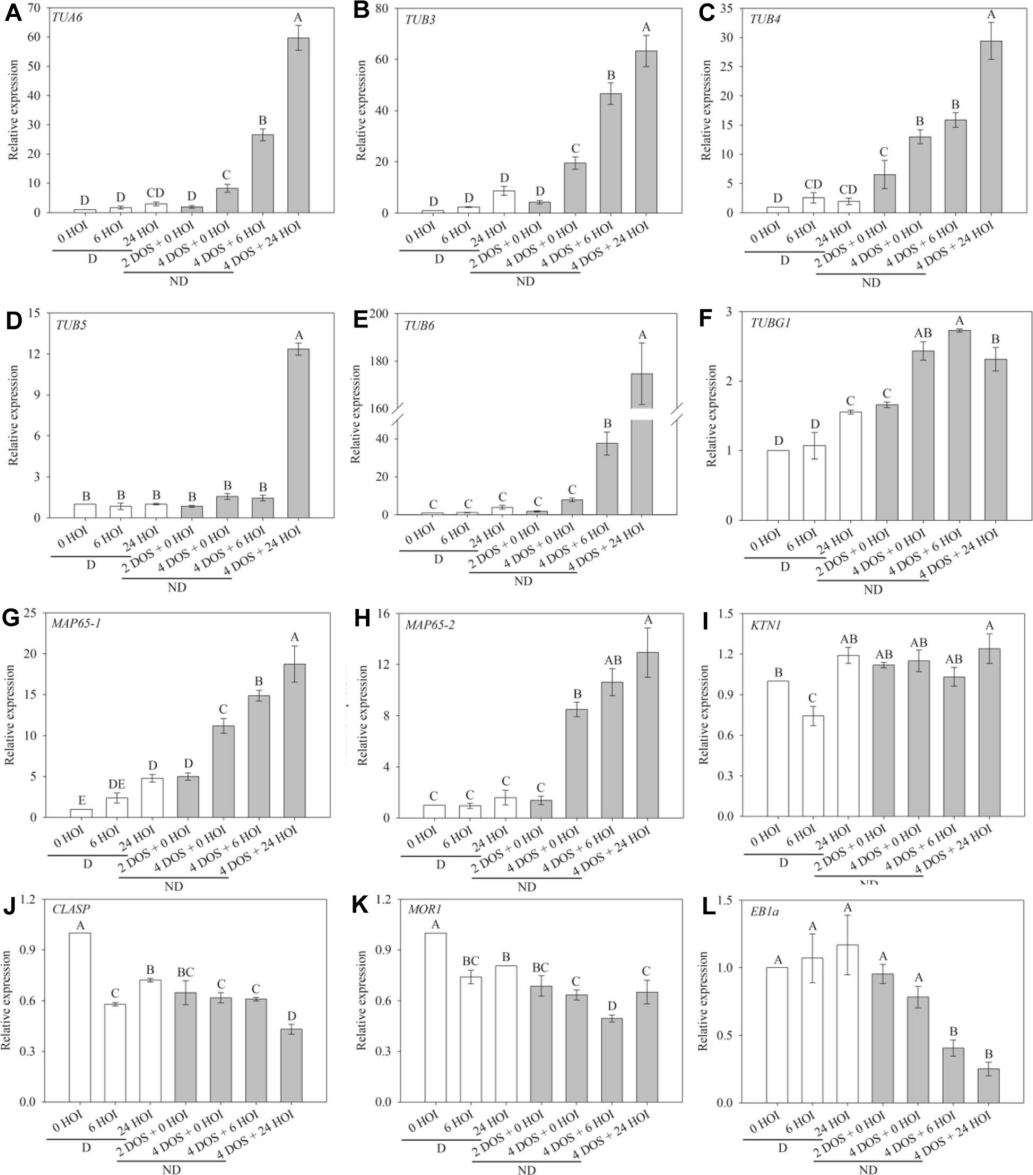
Reorganisation of microtubules during germination is associated with changes in the expression of microtubule-related genes. Relative expression of *TUA6* (**a**), *TUB3* (**b**), *TUB4* (**c**), *TUB5* (**d**), *TUB6* (**e**), *MAP65-1* (**f**), *MAP65-2* (**g**) and *EB1a* (**h**) genes in dormant and non-dormant *Arabidopsis* seeds. HOI, hours of imbibition at 25 °C in the dark; DOS, days of stratification at 4 °C in the dark. 0 HOI corresponds to dry dormant seeds and is used as a reference condition. Different letters indicate significant differences among samples (one-way ANOVA). Values are the means ± s.e. of 3 independent biological replicates with 3 technical replicates each

To test that hypothesis, we followed the changes in expression of key genes likely to be involved in microtubule organisation and synthesis by qRT-PCR in Col-0 seeds (Fig. 3). The expression level of α- and β-tubulin genes was low in dormant dry seeds and during their subsequent imbibition at 25 °C whereas cold stratification progressively induced their upregulation (Fig. 3a–e). *TUB6* expression, noticeably, was dramatically stimulated at 24 HOI (Fig. 3e). The expression of *TUBG1*, coding for γ-tubulin, followed a similar trend but in a much lower extend (Fig. 3f). The expression of the two main microtubule-associated proteins involved in microtubule bundling, *MAP65-1* and *MAP65-2* [27], followed the same pattern as tubulin genes (Fig. 3g, h). Katanin (KTN1) is a microtubule-severing protein which contributes to microtubule self-organisation and to embryo and seed formation in *Arabidopsis* [28]. Its expression remained unchanged whatever the seed sample (Fig. 3i). *CLASP* encodes a microtubule-associated protein which promotes CMT self-organisation by stabilising their polymerisation at cell edges [29, 30]. MICROTUBULE ORGANIZATION 1 (MOR1) is a member of the highly conserved MAP215 family of microtubule-associated proteins that contribute to microtubule polymerisation [31, 32]. The expression of both *CLASP* and *MOR1* genes was lower in imbibed seeds than in dry seeds and was not affected by the dormancy status (Fig. 3j, k). End binding 1a (*EB1a*) stabilises microtubule plus ends [33]. Its expression was high in dry and imbibed dormant seeds, but it decreased markedly during stratification and imbibition of non-dormant seeds (Fig. 3l). Altogether, this suggests that the release of dormancy by cold stratification induces progressive changes in microtubule behaviour during germination, notably through transcriptional induction of tubulin and bundling factors (like MAP65), while imbibition alone is not sufficient to trigger the expression of the complete set of microtubule regulators.

The presence of mRNA is only an indirect indication of gene function. To further check whether protein translation followed the same trend, we explored previously obtained data related to changes in translatome, i.e. transcripts associated with polysomes and undergoing active translation, in dormant and non-dormant seeds at 16 and 24 h of imbibition at 25 °C (Additional file 3: Table S1) [34]. In dormant seeds, proteins involved in microtubules nucleation, such as γ-tubulin, were translated, but proteins involved in microtubule dynamics, such as tubulin (essential for microtubule polymerisation) or MAP65 (essential for microtubule bundling), were likely in limited number (Additional file 3: Table S1). Conversely, upon the release of dormancy, the corresponding mRNAs were translated, matching the formation of organised CMT arrays. CLASP, MOR1 and KTN1 mRNAs were not found in the translatome. Incidentally, the translatome also revealed major modifications in the profile of cell wall modifiers, including promoters of wall weakening, such as pectin lyases and expansins. We could also confirm the induction of tubulin α expression at the protein level by western blot (Additional file 4: Figure S3): α-tubulin accumulated in stratified seeds only, prior to radicle protrusion.

Altogether, these results provide a scenario in which water imbibition changes the stress pattern before the CMTs are able to respond, because the microtubule dynamics protein network is not fully operational yet.

### Hormone-dependent modulation of dormancy regulates microtubule organisation

**Fig. 4:**
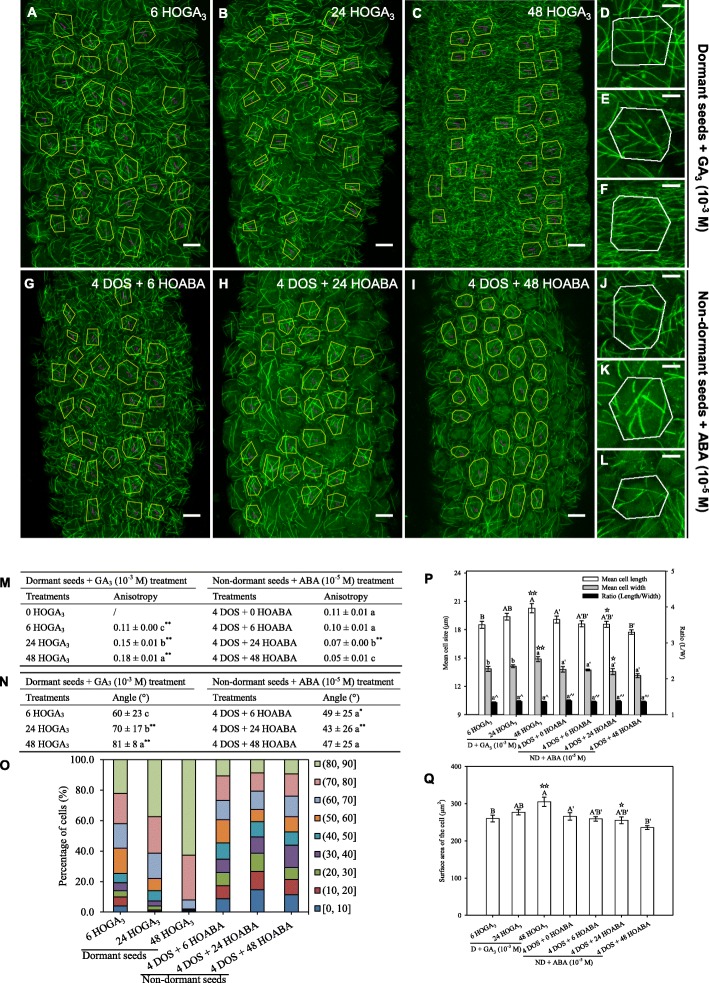
Gibberellic acid and abscisic acid modify microtubule organisation in dormant and non-dormant seeds. Microtubule organisation was assessed in embryos of dormant and non-dormant (4 days of stratification (DOS)) *p35S::GFP-MBD* seeds imbibed for 6, 24 and 48 h (HOI) in the presence of gibberellic acid (GA_3_, 1 mM) (**a**–**f**) and abscisic acid (ABA, 10 μM) (**g**–**l**), respectively. Microtubule average orientation (indicated by purple lines inside the cells) was obtained using FibrilTool and is shown in the regions of interest (cells excluding anticlinal walls) that are delineated with yellow lines (**a**–**c** and **g**–**i**). **d**–**f**, **j**–**l** Microtubule organisation at the level of individual cells from dormant seeds imbibed for 6, 24 and 48 h with GA_3_ and from non-dormant seeds imbibed for the same duration with ABA, respectively (scale bars, 10 μm in images of multicellular hypocotyl and 5 μm in images of individual cells). Values of anisotropy (**m**) and angles (**n**) of CMT arrays were calculated for the same samples. In (**m**, **n**) different letters indicated significant differences within the same column (one-way ANOVA), and asterisks indicate significant difference between non-dormant seeds and dormant seeds at every the same time point (*t* test, *P < 0.05, **P < 0.01). **o** Angle distribution (expressed in %) among cells of the various samples is shown for every sample. Changes in cell shape were evaluated by measuring the cell length and width (**p**) and area (**q**), in which different letters indicate significant differences among all samples of GA_3_ or ABA treatments (one-way ANOVA), and stars indicate significant difference between non-dormant seeds and dormant seeds at every the same time point (*t* test, ^☆^*P* < 0.05, ^☆☆^*P* < 0.01). Values of CMT array angles (**n**) were expressed as means ± s.d. while values of CMT array anisotropy (**m**) and cell shape (**p**, **q**) were expressed as means ± s.e. Five biological replicates were analysed, and 30 cells from each replicate were used to calculate the indicated values

If true, our hypothesis should be verified whether dormancy is released by cold or by other means. In particular, upon treatment with GA, which is also known to accumulate during cold stratification [35], seeds became able to fully germinate at 25 °C within 4–5 days, without any cold stratification treatment (Additional file 1: Figure S1c). When treated with GA_3_, the induction of germination could be detected by changes in cell shape and size (Fig. 4p, q). Microtubules progressively became more ordered in the *p35S::GFP-MBD* line, and this was associated with an increase in CMT array anisotropy (from 0.11 after 6 h of GA_3_ treatment to 0.18 after 48 h of GA_3_ treatment) and reduced standard deviation of CMT angles (from 60 ± 23° after 6 h of GA_3_ treatment to 81 ± 8° after 48 h of GA_3_ treatment) (Fig. 4m–o). The impact of the release of dormancy on CMTs is thus not strictly dependent on cold, but rather seems a generic feature of the early steps of germination.

Conversely, germination can be inhibited by ABA. When using this treatment on stratified seeds (i.e. non-dormant seeds), we observed that, during seed imbibition, cell length, width and surface area did not evolve significantly (Fig. 4p, q). Consistently, CMTs remain disorganised in the *p35S::GFP-MBD* line (Fig. 4g–i, j–l). In fact, we could even detect a decrease of CMT array anisotropy (from 0.11 after 6 h of ABA treatment to 0.05 after 48 h of ABA treatment) when stratified seeds were imbibed in the presence of ABA (Fig. 4m). Accordingly, the standard deviation of CMT array angles remained high during seed imbibition (49 ± 25° at 6 h and 47 ± 25° at 48 h after ABA treatment, Fig. 4n, o). This confirms that imbibition is not sufficient to trigger a change of CMT behaviour and that dormancy needs to be released for CMT arrays to become more ordered.

### Expression of microtubule regulators during the release of dormancy is under hormonal control

**Fig. 5:**
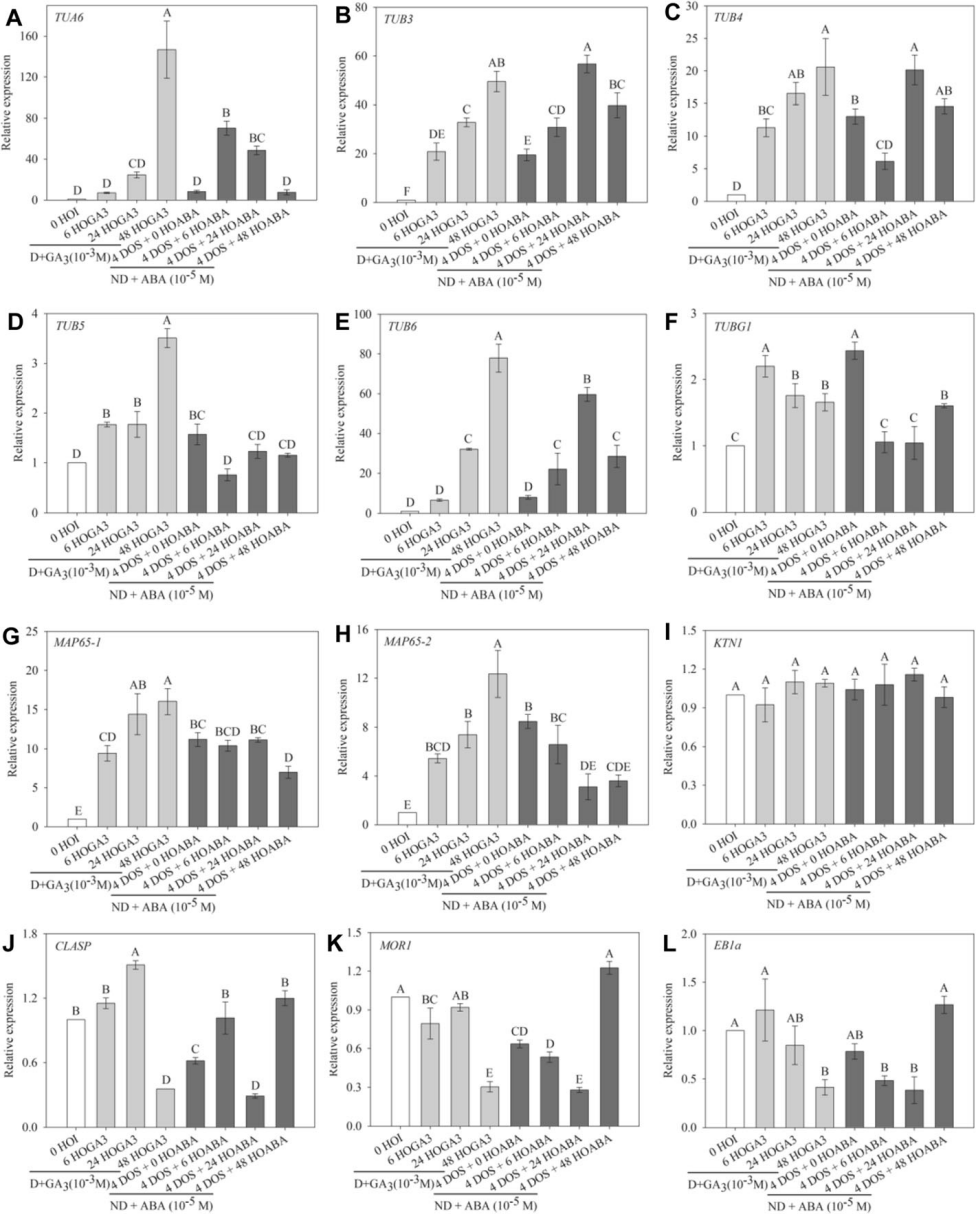
Transcriptional regulation of microtubule organisation by GA_3_ and ABA. Relative expression of *TUA6* (**a**), *TUB3* (**b**), *TUB4* (**c**), *TUB5* (**d**), *TUB6* (**e**), *MAP65-1* (**f**), *MAP65-2* (**g**) and *EB1a* (**h**) genes in dormant and non-dormant *Arabidopsis* seeds. HOI, hours of imbibition at 25 °C in the dark; DOS, days of stratification at 4 °C in the dark. 0 HOI corresponds to dry dormant seeds and is used as a reference condition. Dormant seeds (**d**) were imbibed at 25 °C in the presence of gibberellic acid (GA_3_) for 6, 24 and 48 h (6 HOGA_3_, 24 HOGA_3_ and 48 HOGA_3_, respectively). Stratified non-dormant seeds (ND) were imbibed at 25 °C on water (0 HOABA) and in the presence of abscisic acid (ABA) for 6, 24 and 48 h (6 HOABA, 24 HOABA and 48 HOABA, respectively). Different letters indicate significant differences among samples (one-way ANOVA). Values are the means ± s.e. of 3 independent biological replicates with 3 technical replicates each

To further confirm our hypothesis, we also checked the expression of some genes involved in microtubule dynamics after the hormone treatments. *TUA6*, *TUB5* and *TUB6* gene expression increased in the presence of GA_3_, and this was not the case in the presence of ABA (Fig. 5a, d, e). Note that *TUB3* and *TUB4* gene expression tended to increase during seed imbibition either in the presence of GA_3_ or ABA (Fig. 5b, c). *TUBG1* expression was slightly stimulated in the presence of either ABA or GA_3_ (Fig. 5f). The expression of *MAP65-1* and *MAP65-2* increased during imbibition of dormant seeds in the presence of GA_3_ (Fig. 5g, h), when it remained unchanged or decreased, respectively, in the case of non-dormant seeds imbibed with ABA (Fig. 5g, h). The expression of *KTN1* remained unchanged whatever the seed treatment (Fig. 5i). GA_3_ treatment induced a downregulation of *CLASP*, *MOR1* and *EB1a*, after 48 h of imbibition of dormant seeds (Fig. 5j–l). In the presence of ABA, *CLASP*, *MOR1* and *EB1a* expression also started to decrease after 6 and 24 h of imbibition but abruptly increased at 48 h (Fig. 5j–l). However, the amplitude of variation of expression of these 3 genes was very weak (always lower to 2-fold). The role of the hormonal balance on CMT organisation was confirmed by studying the expression of the same genes during germination of mutants altered in GA or ABA signalling, using publicly available transcriptomic data (Additional file 5: Figure S4). In ABA signalling mutants, and particularly in *abi3-4*, *abi4-11* and *abi5-7*, genes involved in microtubule organisation and synthesis were overexpressed during seed imbibition, excepted for *TUA6*, *KTN1* and *MOR1*. In the seeds of the GA-deficient mutant *ga1-3*, only *TUA6* and *MAP65-2* were downregulated, whereas the expression of the other genes did not vary. There were no data available concerning the expression of *EB1a* in mutant seeds.

## Discussion

**Fig. 6:**
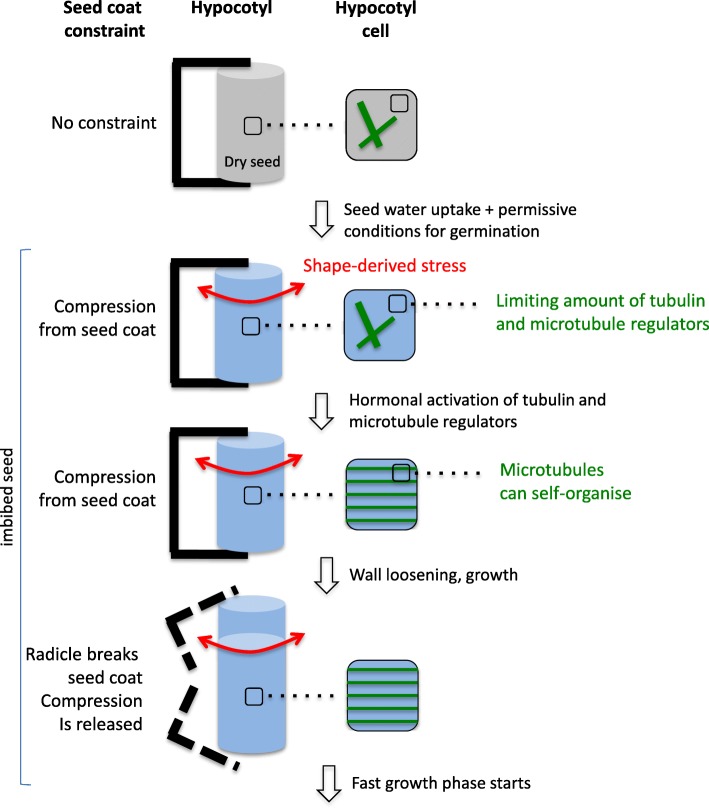
Model: microtubule self-organisation during seed germination and symmetry breaking in the growing hypocotyl. This model explains how dormancy release allows self-organisation of cortical microtubules when conditions are permissive for germination. See text for explanations

The role of biomechanics in seed germination is attracting increasing attention [2, 36], notably to reveal the role of endosperm weakening as a regulator of radicle protrusion [36]. Here, we report the organisation of CMTs as they appear during seed imbibition and germination, and we demonstrate that the release of dormancy triggers their self-organisation into parallel arrays. We show that the dynamics of CMT leading to their transverse orientation in the hypocotyl area of the embryonic axis, i.e. where cell expansion takes place [3, 4], is tightly associated with radicle protrusion and is regulated by the dormancy status. To observe CMT, we had to remove the seed coat. Such perturbation may induce artefactual CMT behaviour. Based on the data obtained in shoot apical meristems and cotyledons, it appears that CMT can retrieve a normal behaviour within 6 to 16 h [23, 37]. Although we cannot formally exclude a contribution of this initial perturbation, our observations were conducted for several days, and thus, it is unlikely that the observed CMT behaviour in the long term is caused by this initial perturbation. Furthermore, the comparison of CMT behaviour between dormant and non-dormant seeds offers an internal control for the effect of seed coat removal on CMT organisation. We indeed observed that bundling disappears upon the release of dormancy and remains unchanged when dormancy is maintained, which offers an intrinsic control to make sure that the degree of MT bundling is not only the result of seed coat removal. The ability of seeds to germinate relies on a rapid reorganisation of CMTs, initiated as early as 6 h of imbibition (Figs. 1, 2 and 5). This process is not strictly related to the changes in turgor pressure occurring when a dry quiescent seed gets imbibed: 4 days of stratification or imbibition of dormant seeds did not markedly modify CMT organisation. This finding was confirmed by using ABA, which blocked germination, and GA_3_, which alleviated dormancy: both hormones had antagonistic effects on CMT organisation (see Fig. 5). Beyond tensile stress, the effect of the plant hormones ABA and GAs on microtubule organisation has already been demonstrated but often in other contexts than germination [14, 38, 39]. In these reports, ABA treatment usually results in a predominance of longitudinal CMTs, whereas GAs induce predominance of transverse CMTs. Da Silva et al. [40] reported that exogenous ABA could inhibit transverse organisation of CMT in imbibed coffee seeds. CMT reorganisation in imbibed seeds would therefore require a state of competence controlled by both exogenous (i.e. appropriate temperature) and endogenous (i.e. dormancy status and hormones) factors, allowing CMTs to form multiple arrays. As they become able to self-organise, CMTs also become sensitive to tensile stress, and their consistent transverse orientation then fuels subsequent directional cell elongation (Fig. 6).

Using an automated confocal micro-extensometer, CMTs were shown to become transverse in compressed hypocotyls [13]. Our results are consistent with these findings, albeit in the natural context of an expanding embryo where the constraint is brought by the stiff envelope of the seed and the endosperm layer and not by a micro-extensometer. We cannot exclude that other factors than mechanical stress only could explain the transverse orientation of CMTs in germinating embryos. For instance, cell shape is a well-known factor that biases CMT orientation. In particular, because of their intrinsic stiffness, CMTs may align so as to minimise their bending energy, and thus preferentially align along the flattest part of the cell. This steric constraint has been proposed to explain why microtubules can align with the longitudinal axis of the cells in silico [41], in confined cells [42, 43] and in tissues undergoing growth arrest [44]. Conversely, microtubule regulators, such as CLASP, have been proposed to help microtubule bend at cell edges [30]. In the germinating embryos, changes in cell shape are minimal, cell shapes are rather isotropic and adjacent cells do not have consistent shapes. Therefore, cell shape alone cannot explain the consistent alignment of CMTs in hypocotyl cells. Hormone gradients could also provide a directional cue that could explain the consistent alignment of CMTs. Such gradients have been shown recently to play a critical role in the completion of germination [45]. However, we observed that CMTs became transverse over the whole length of the hypocotyl, i.e. without a clear gradient, which is consistent with a response to an instantaneous and global compression event. We propose that the impact of hormones on CMTs might rather be indirect, through the expression of microtubule regulators, giving them the competence to self-organise, and through their impact on cell wall properties and/or turgor pressure, to affect mechanical stress levels. As long as the seed envelope remains intact, embryo cells would be mechanically protected despite their turgor pressure building up, whereas upon seed envelope breakdown, aligned CMTs would be required to reinforce cell walls through cellulose synthesis.

Gene expression analysis allowed us to show a transcriptional regulation of CMT reorganisation during germination (see Figs. 3 and 6). Dynamics of tubulin accumulation during seed germination has been poorly studied so far. Such studies, mostly based on β-tubulin immunodetection, have shown that tubulin was present in low amount in dry seeds and that it accumulated during seed stratification and germination [8, 46–48]. Yet, microtubule disorganisation following oryzalin treatment does not interfere with seedling germination in *Medicago truncatula* [49]. Microtubules may not be absolutely required for germination, at least at the early stages. Patterns of cell elongation do not necessarily require microtubules, as shown in meristems [10] and hypocotyls [50]. Furthermore, the absence of microtubules may even weaken the cell wall and speed up cell growth, as shown in shoot meristems [51].

Our results show that CMT reorganisation, which is associated with seed germination, is accompanied by an overexpression of genes coding for α-, β- and γ-tubulin, namely *TUA*6 and *TUB3*, *TUB4*, *TUB5*, *TUB6* and *TUBG1* (Figs. 3a–f and 5a–f). Our results echo previously published proteome of seed germination in *Arabidopsis* [52, 53]. We also show that α-tubulin is detectable in dry dormant seeds but that it accumulates dramatically at the onset of radicle protrusion only (Additional file 4: Figure S3). In contrast, *EB1a* showed the inverse expression pattern since it decreased when seeds germinated (Figs. 3h and 6h). However, there was not a clear cut relationship with radicle elongation and expression of tubulin and *EB1a* genes since they were not fully repressed or induced by ABA, respectively (Fig. 6a–e, h), which suggests that tubulin accumulation and EB1a may be regulated by other factors than the sole ABA/GA balance. As *EB1* overexpression induces the formation of microtubule bundles [54], the relatively high level of *EB1* in imbibed dormant seeds might also contribute to explain why CMTs form few thick bundles at that stage. Interestingly, the expression of both CLASP and MOR1 was relatively stable in dormant and non-dormant seeds. As both CLASP and MOR1 also relate to microtubule plus-end tracking proteins like EB1 [55, 56], this class of regulators may rather be under post-transcriptional control during germination. The profile of MAP65 proteins matches the dynamics of CMT bundles (Figs. 3f, g and 5f, g). Their expression increased when seeds germinated but was repressed in the presence of ABA. MAP65s are key regulators of cortical CMT organisation because they control CMT bundling [57] thus promoting axial growth of roots and hypocotyl cells in *Arabidopsis* [20, 58]. Our data therefore strongly suggest that hormones participate in CMT reorganisation through the transcriptional activation of tubulin and CMT regulators. It is worth noting that the analysis of expression of these former genes in ABA- and GA-mutant seeds confirmed this finding (Additional file 4: Figure S3). Lastly, the translatome data (Additional file 3: Table S1) provide further molecular insights: translation of protein involved in cell wall remodelling did not occur upon imbibition, but only later on as cells initiated their shape changes. In contrast, the association of transcripts involved in CMT organisation with polysomes occurred at an early time point of imbibition, when seeds were engaged in the germination process, but before detectable cell shape changes.

Our results thus add an additional layer of complexity in the control of growth anisotropy in the hypocotyl during seed germination, along the possible following scenario (Fig. 6): (1) seed water uptake pressurises cells and tissue, but microtubule organisation is first hindered by the limiting amount of tubulin and regulators; (2) release of dormancy and changes in hormonal balance promotes the expression of tubulin and microtubule regulators, thereby allowing their organisation in parallel arrays; (3) shape-derived tensile stress dominates and prescribe transverse CMT orientations, while wall loosening increases stress levels in a feedback loop; and (4) the radicle breaks the envelope.

## Conclusion

We report the dynamic changes in CMT organisation during seed germination. We show that CMTs first appear in *Arabidopsis* development as few thick bundles, and later on switch to parallel and transverse arrays when the seed germinates, following the predicted tensile stress pattern that is prescribed by tissue pressurisation, compression and cylindrical shape. We also show that the release of dormancy is associated with the expression and translation of a subset of microtubule regulators, thereby triggering the ability of CMTs to organise in organised arrays during germination and to control growth anisotropy. These findings reveal an additional component in the regulation of seed germination by dormancy. As a prospect, taking into account the mechanical constraints applied by the seed coat on the embryo might also shed a new light on the developmental programme behind germination.

## Methods

### Plant material, treatments and germination assays

*Arabidopsis* (*Arabidopsis thaliana*) seeds of the wild-type Col-0 (expression assays, translatome) and of the microtubule-binding domain marker line *p35S::GFP-*MBD [59] in the Wassilewskija Ws-4 background [10] and β-tubulin marker line *p35S::GFP-TUB6* (in Columbia Col-0 background) [60] were used in the experiments. Freshly harvested seeds were dormant and stored at − 20 °C to preserve their dormancy. Germination assays were carried out by placing 3 replicates of 100 seeds on a single-layer filter paper on the top of cotton wool moistened with deionised water, or with the solutions indicated in the text (10^−3^ M GA_3_ or 10^−5^ M ABA), in 9-cm Petri dishes at 25 °C in darkness. Stratification was performed by placing seeds on a filter paper on the top of a cotton wool moistened with deionised water at 4 °C for 2 or 4 days in the darkness, before their transfer to 25 °C. Germination was evaluated daily, and a seed was considered as germinated when the radicle protruded through the testa.

Because the *p35S::GFP-MBD* and *p35S::GFP-TUB6* constructs used in this study may affect cell physiology, we confirmed that seeds of both lines displayed dormancy [6]: they were unable to germinate at 25 °C in the darkness, and cold stratification progressively released dormancy (Additional file 1: Figure S1). Four days of stratification were necessary to allow full germination of seeds of both lines at 25 °C (Additional file 1: Figure S1). The experimental time points used for the experiments in this manuscript were chosen based on these germination curves (arrows on Additional file 1: Figure S1).

### Sample preparation and image acquisition by confocal microscopy

After the various conditions of imbibition mentioned in the text, embryos were extracted from seeds with a forceps under a binocular. Isolated embryos were immediately mounted in a distilled water medium in a glass chamber (2 mm thickness) covered with a coverslip and observed by confocal microscopy.

For Calcofluor staining, isolated embryos were immersed in 0.1% (w/v) fluorescent brightener 28 for 30 min at 25 °C in darkness, and then they were washed 3–4 times with MilliQ water. Stained embryos were mounted in MilliQ water between a glass slide and a coverslip and immediately observed by confocal microscopy using epifluorescent UV irradiation.

Three-dimensional (3D) fluorescence images of hypocotyl cells were acquired on a Leica TCS SP5 confocal microscopy equipped with Hybrid Detectors and Acousto-Optical Tunable Filters/Acousto-Optical Beam-Splitters (AOTF/AOBS) for creating spectral emission windows. Argon laser excitation wavelength at 488 nm and an emission window of 495–580 nm were used for capturing the GFP signal. The brightener fluorescence signal was measured at an excitation of 405 nm with an emission window of 412–480 nm. Either a × 40 oil immersion lens (N.A. of 1.25, free working distance of 0.1 mm) for GFP images or a × 20 oil immersion lens (N.A. of 0.70, free working distance of 0.17 mm) for images of brightener fluorescence was used.

Confocal z-stacks of embryos at different time points were processed by converting μm (a slice interval in z-step size), which gave a voxel size of 0.152 μm × 0.152 μm × 0.168 μm (*xyz*) and an image size of 155.00 μm × 155.00 μm (*xy*). Optical slices of 8-bit depth 3D images were captured using Leica Application Suite-Advanced Fluorescence (LAS-AF) software.

### Visualisation and image analysis

Confocal z-stacks of embryos were processed by converting 3D image to a 2D *x*-*y* plane image with maximum intensity projection, and brightness/contrast of images was edited using “Brightness/Contrast” tool in the ImageJ software. Thirty cells of each embryo (5 embryos for each sample) were used to quantify the anisotropy and average orientation of microtubule with FibrilTool, an ImageJ plug-in, by drawing the region of interest (ROI) in 2D *x*-*y* plane images, according to the procedure from [21]. Evaluation of anisotropy was based on the score, with 0 for no order (purely isotropic arrays) and 1 for perfect order. Average orientation of microtubule was the angle with respect to the elongation axis of the cell (*y*-axis of the image).

The outline of 30 cells in the hypocotyl of each embryo was drawn in optical slices of 3D images, and the mean cell length and width were measured by the ImageJ software.

### RNA extraction and qRT-PCR

A 60-mg aliquot of seeds was ground in liquid nitrogen with insoluble PVP, and total RNA was extracted with a hot phenol procedure according to [61]. Purified RNA (2 μg) was reverse transcribed and amplified to synthesise cDNA using the RevertAid Reverse Transcriptase (Thermo Scientific), and quantitative real-time PCR was performed using Mastercycler ep Realplex (Eppendorf). Primer sequences are listed in Additional file 6: Table S2. Relative expression of genes was calculated with the 3 reference genes *At5g53560*, *At4g26410* and *At4g34270* as described by [6]. Results presented are the means ± s.e. of 3 biological replicates.

### Immunoblotting

Seeds (30 mg FW) were ground in liquid nitrogen in 1 ml of MEB buffer (50 mM Hepes-KOH (pH 7.5), 5 mM EDTA (pH 8.0), 5 mM EGTA (pH 8), 25 mM sodium fluoride, 1 mM sodium orthovanadate, 50 mM β-glycerophosphate, 75 mM NaCl, 20% glycerol, 0.1% Triton X-100, 5 mM DTT, 1× protease inhibitor cocktail). The homogenate was then centrifuged at 14,000*g* for 45 min at 4 °C. Proteins were denaturated in 40 μl of Laemmli loading buffer and separated by 10% sodium dodecyl sulfate-polyacrylamide gel electrophoresis (SDS-PAGE). After separation, the proteins were transferred electrophoretically (20 V, 35 min) onto nitrocellulose using a Trans-Blot semi-dry system (BioRad), blocked with 5% (w/v) non-fat dry milk and hybridised with α-tubulin antibody (Agrisera, no. AS10680) at 1:5000 dilution in 50 mM Tris, 150 mM NaCl, 0.1% (v/v) Tween 20 and 5% (w/v) non-fat dry milk. After washing 3 times with PBS 1× and 1% (w/v) non-fat dry milk, the membranes were incubated with secondary anti-rabbit IgG (Sigma, A6154) at a 1:1000 dilution for 1 h in PBS 1× and 1% (w/v) non-fat dry milk. Proteins were detected by chemiluminescence (Amersham ECL Prime Western Blotting Detection Reagent).

### Statistical analysis

Significant differences between treatments were determined by analysis of one-way variance (ANOVA) at a significance threshold of 5% and 1% using SPSS Statistics software (version 17.0). For comparison of significance between dormant and non-dormant seeds, water and GA_3_ imbibition on dormant seeds, water and ABA imbibition on non-dormant seeds, statistical analysis was performed using Student’s *t* test (*P* < 0.05 and *P* < 0.01). Graphs were created by the SigmaPlot software (version 10.0).

## Supplementary information

**Additional file 1: Figure S1.** Germination of p35S::GFP-MBD and p35S::GFP-TUB6 seeds Germination was performed at 25 °C in darkness on water (a, b) or on a solution of gibberellic acid (GA3) or abscisic acid (ABA) (c). DOS, days of stratification. Arrows indicate the time points of microtubule visualization. (PPTX 62 kb)

**Additional file 2: Figure S2.** Overview of the experimental model. Overview of the experimental model. A, Anatomy of a whole Arabidopsis embryo extracted from an imbibed seed and stained using calcofluor (scale bar, 20 μm). B, Localisation of cortical microtubules in different areas of Arabidopsis embryo using p35S::GFP-MBD. Embryos were extracted from germinating seeds imbibed for 48 h at 25°C in darkness in the presence of 1 mM GA_3_ (scale bars, 20 μm). (PPTX 1668 kb)

**Additional file 3: Table S1.** Shortlist of microtubule-related genes in the translatome from dormant and non-dormant seeds

**Additional file 4: Figure S3.** Immunodetection of α tubulin. A 5 μg aliquot of total protein was loaded on each lane and α-tubulin was detected with α-tubulin antibody in dormant and non-dormant *Arabidopsis* seeds. HOI, hours of imbibition at 25°C in the dark; DOS, days of stratification at 4°C in the dark. (PPTX 935 kb)

**Additional file 5: Figure S4.** Expression of tubulin and tubulin regulators genes in mutant seeds development. Expression of *TUB3, TUB4, TUB5, TUB6, TUA6, MAPD65-1, MAP65-2, CLASP, KTN1, MOR1* and *TBG1* in GA and ABA mutant seeds (indicated in figures). Data obtained through the Arabidopsis eFP browser at bar.toronto.ca. (PPTX 11238 kb)

**Additional file 6: Table S2.** Primer sequences used for qRT-PCR.

## Data Availability

Transcriptome and translatome data analyzed in this article are deposited at the Gene Expression Omnibus (http://www.ncbi.nlm.nih.gov/geo/; accession no. GSE61809) and were previously published by [34]. The raw imaging data supporting the conclusions of this article are available from Figshare (10.6084/m9.figshare.12017181) [62]. The material and datasets used and/or analysed during the current study are available from the corresponding author on reasonable request.
