# Supplementary material for: Microtubule self-organisation during seed germination in Arabidopsis
Source: BMC Biol. 2020 Apr 30;18:44. doi: 10.1186/s12915-020-00774-8 (PMC7191766; doi:10.1186/s12915-020-00774-8)
Supplement: Supplementary file 1 — Additional file 1: Figure S1. Germination of p35S::GFP-MBD and p35S::GFP-TUB6 seeds Germination was performed at 25 °C in darkness on water (a, b) or on a solution of gibberellic acid (GA3) or abscisic acid (ABA) (c). DOS, days of stratification. Arrows indicate the time points of microtubule visualization. (PPTX 62 kb) [file 12915_2020_774_MOESM1_ESM.pptx]

## Slide 1
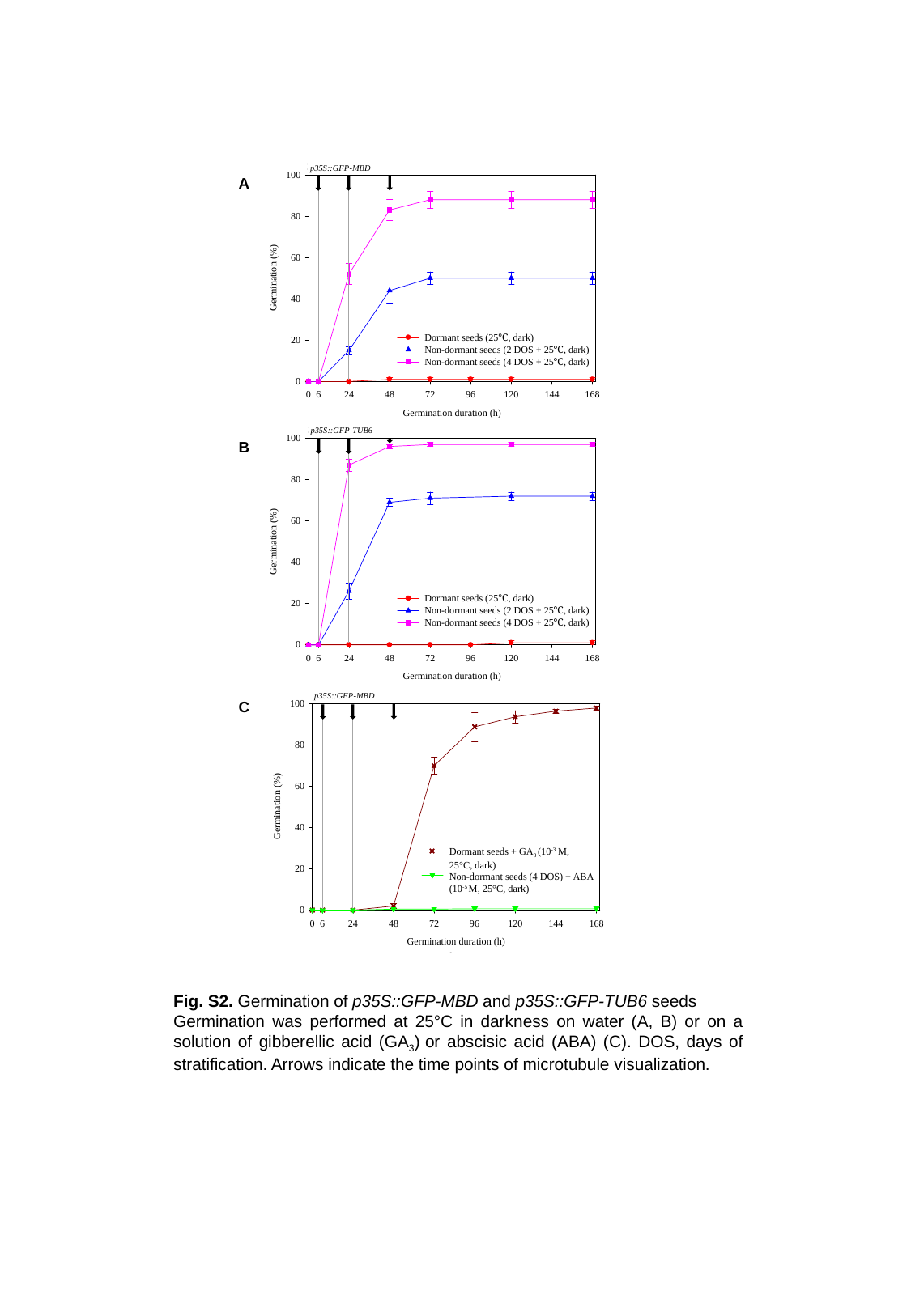

p35S::GFP-MBD
A
p35S::GFP-TUB6
B
p35S::GFP-MBD
C
Dormant seeds + GA3 (10-3 M, 25°C, dark)
Non-dormant seeds (4 DOS) + ABA (10-5 M, 25°C, dark)
Fig. S2. Germination of p35S::GFP-MBD and p35S::GFP-TUB6 seeds
Germination was performed at 25°C in darkness on water (A, B) or on a solution of gibberellic acid (GA3) or abscisic acid (ABA) (C). DOS, days of stratification. Arrows indicate the time points of microtubule visualization.
