# Supplementary material for: Microtubule self-organisation during seed germination in Arabidopsis
Source: BMC Biol. 2020 Apr 30;18:44. doi: 10.1186/s12915-020-00774-8 (PMC7191766; doi:10.1186/s12915-020-00774-8)
Supplement: Supplementary file 2 — Additional file 2: Figure S2. Overview of the experimental model. Overview of the experimental model. A, Anatomy of a whole Arabidopsis embryo extracted from an imbibed seed and stained using calcofluor (scale bar, 20 μm). B, Localisation of cortical microtubules in different areas of Arabidopsis embryo using p35S::GFP-MBD. Embryos were extracted from germinating seeds imbibed for 48 h at 25°C in darkness in the presence of 1 mM GA3 (scale bars, 20 μm). (PPTX 1668 kb) [file 12915_2020_774_MOESM2_ESM.pptx]

## Slide 1
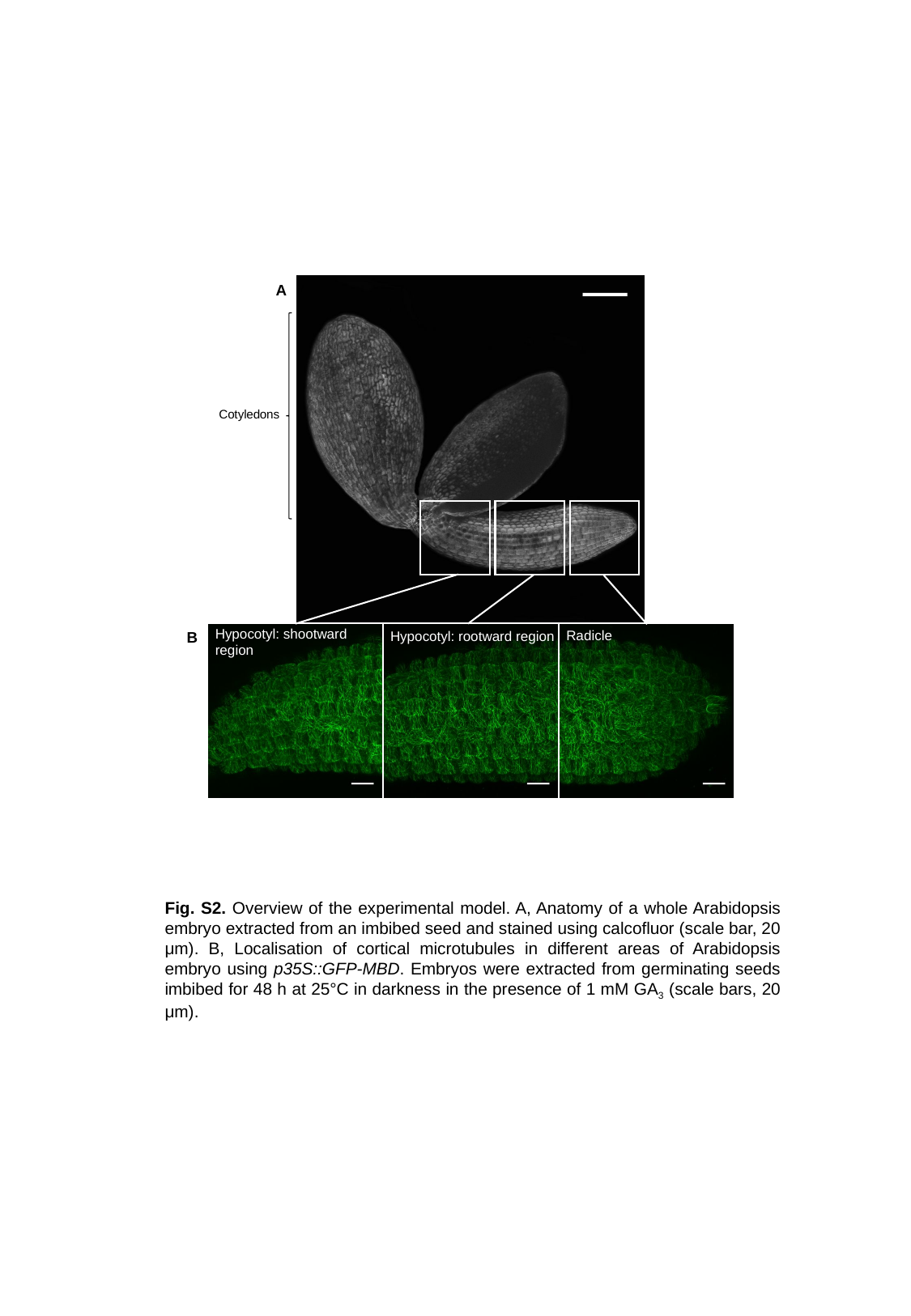

A
Hypocotyl: shootward region
Radicle
Hypocotyl: rootward region
B
Cotyledons
Fig. S2. Overview of the experimental model. A, Anatomy of a whole Arabidopsis embryo extracted from an imbibed seed and stained using calcofluor (scale bar, 20 μm). B, Localisation of cortical microtubules in different areas of Arabidopsis embryo using p35S::GFP-MBD. Embryos were extracted from germinating seeds imbibed for 48 h at 25°C in darkness in the presence of 1 mM GA3 (scale bars, 20 μm).
