# Supplementary material for: Microtubule self-organisation during seed germination in Arabidopsis
Source: BMC Biol. 2020 Apr 30;18:44. doi: 10.1186/s12915-020-00774-8 (PMC7191766; doi:10.1186/s12915-020-00774-8)
Supplement: Supplementary file 3 — Additional file 3: Table S1. Shortlist of microtubule-related genes in the translatome from dormant and non-dormant seeds [file 12915_2020_774_MOESM3_ESM.pdf]

Table S1 Annotations of transcripts differentially abundant in translatome of dormant (D) and non-dormant (ND) Arabidopsis seeds and belonging to the TAIR categories "cell organisation" and "cell wall"

Seeds were imbibed for 16 h or 24 h at 25°C

Ratio corresponds to the relative level of abundance of transcript in the polysomal fraction of D vs ND seeds

TAIR "cell organisation"

D 16 h

Gene ID	Tair Annotation	Ratio
At5g56180	ARP8_ARP8_ATARP8__actin-related protein 8	-1.01
At3g57890	Tubulin binding cofactor C domain-containing protein	-0.91
At3g04710	TPR10__ankyrin repeat family protein	-0.88
At2g21950	SKIP6__SKP1 interacting partner 6	-0.88
At2g42170	Actin family protein	-0.86
At5g37590	Tetratricopeptide repeat (TPR)-like superfamily protein	-0.85
At3g60740	CHO_EMB133_TFC D_TTN1__ARM repeat superfamily protein	-0.83
At1g70140	ATFH8_FH8__formin 8	-0.81
At5g65930	KCBP_PKCBP_ZWI__kinesin-like calmodulin-binding protein (ZWICHEL)	-0.81
At3g05520	Subunits of heterodimeric actin filament capping protein Capz superfamily	-0.79
At1g71790	Subunits of heterodimeric actin filament capping protein Capz superfamily	-0.78
At2g46250	myosin heavy chain-related	-0.77
At2g46910	Plastid-lipid associated protein PAP / fibrillin family protein	-0.74
At5g18580	EMB40_FASS_FASS 2_FS1_GDO_TON2__tonneau 2 (TON2)	-0.73
At3g01890	SWIB/MDM2 domain superfamily protein	-0.71
At4g31340	myosin heavy chain-related	-0.69
At2g42090	ACT9__actin 9	-0.67
At2g38440	ATSCAR2_DIS3_ITB1_SCAR2_WAVE4__SCAR homolog 2	-0.67
At1g18450	ARP4_ATARP4__actin-related protein 4	-0.67
At2g46225	ABIL1__ABI-1-like 1	-0.65
At1g06530	PMD2__Tropomyosin-related	-0.64
At4g16340	SPK1__guanyl-nucleotide exchange factors;GTPase binding;GTP binding	-0.63
At3g61650	TUBG1__gamma-tubulin	-0.62
At5g53400	BOB1__HSP20-like chaperones superfamily protein	-0.62
At5g18680	AtTLP11_TLP11__tubby like protein 11	-0.61
At5g07840	PIA1__Ankyrin repeat family protein	-0.60
At1g24460	TNO1__unknown protein; FUNCTIONS IN: molecular_function unknown; INVOLVED IN: bio	-0.59
At3g16120	Dynein light chain type 1 family protein	-0.59
At3g26080	plastid-lipid associated protein PAP / fibrillin family protein	-0.58
At3g44730	AtKIN14h_ATKP1_KP1__kinesin-like protein 1	-0.57
At1g15730	Cobalamin biosynthesis CobW-like protein	-0.57
At2g41370	BOP2__Ankyrin repeat family protein / BTB/POZ domain-containing protein	-0.57
At5g07740	actin binding	-0.56
At1g04780	Ankyrin repeat family protein	-0.56
At5g65460	KAC2_KCA2__kinesin like protein for actin based chloroplast movement 2	-0.55

ND 16 h

Gene ID	Tair Annotation	Ratio
At3g47430	PEX11B__peroxin 11B	0.53
At5g54670	ATK3_KATC__kinesin 3	0.57
At3g45850	P-loop containing nucleoside triphosphate hydrolases superfamily protein	0.58
At3g61650	TUBG1__gamma-tubulin	0.59
At5g45110	ATNPR3_NPR3__NPR1-like protein 3	0.59
At4g14147	ARPC4__protein binding	0.60
At2g41740	ATVLN2_VLN2__villin 2	0.60
At5g12250	TUB6__beta-6 tubulin	0.64
At1g47200	WPP2__WPP domain protein 2	0.66
At5g59370	ACT4__actin 4	0.69
At1g04820	TOR2_TUA4__tubulin alpha-4 chain	0.70
At5g14230	CONTAINS InterPro DOMAIN/s: Ankyrin repeat-containing domain (InterPro:IPR020683), An	0.73
At5g65020	ANNAT2__annexin 2	0.78
At5g06680	ATGCP3_ATSPC98_GCP3_SPC98__spindle pole body component 98	0.79
At5g62500	ATEB1_ATEB1B_EB1B__end binding protein 1B	0.79
At4g26760	MAP65-2__microtubule-associated protein 65-2	0.84
At2g29550	TUB7__tubulin beta-7 chain	0.85
At4g14960	TUA6__Tubulin/FtsZ family protein	0.86

At5g23860	TUB8__tubulin beta 8	0.88
At5g33300	chromosome-associated kinesin-related	0.95
At3g47690	AtEB1a_ATEB1H2_EB1a__microtubule end binding protein EB1A	0.97
At3g24530	AAA-type ATPase family protein / ankyrin repeat family protein	0.98
At5g62700	TUB3__tubulin beta chain 3	0.98
At2g38750	ANNAT4__annexin 4	1.05
At4g34970	ADF9__actin depolymerizing factor 9	1.08
At3g53750	ACT3__actin 3	1.09
At1g75780	TUB1__tubulin beta-1 chain	1.15
At3g04630	WDL1__WVD2-like 1	1.18
At3g12110	ACT11__actin-11	1.27
At3g56480	myosin heavy chain-related	1.27
At1g20010	TUB5__tubulin beta-5 chain	1.30
At2g16700	ADF5_ATADF5__actin depolymerizing factor 5	1.42

D 24 h

Gene ID	Tair Annotation	Ratio
	N/A	

ND 24 h

Gene ID	Tair Annotation	Ratio
At5g28646	WVD2__TPX2 (targeting protein for Xklp2) protein family	0.58
At2g01750	ATMAP70-3_MAP70-3__microtubule-associated proteins 70-3	0.59
At3g26070	Plastid-lipid associated protein PAP / fibrillin family protein	0.59
At1g47750	PEX11A__peroxin 11A	0.59
At1g20570	Spc97 / Spc98 family of spindle pole body (SBP) component	0.61
At5g65460	KAC2_KCA2__kinesin like protein for actin based chloroplast movement 2	0.62
At5g65860	ankyrin repeat family protein	0.62
At1g14840	ATMAP70-4_MAP70-4__microtubule-associated proteins 70-4	0.63
At5g02370	ATP binding microtubule motor family protein	0.65
At3g53750	ACT3__actin 3	0.65
At3g23280	XBAT35__XB3 ortholog 5 in Arabidopsis thaliana	0.66
At5g57740	XBAT32__XB3 ortholog 2 in Arabidopsis thaliana	0.66
At3g45850	P-loop containing nucleoside triphosphate hydrolases superfamily protein	0.67
At4g26760	MAP65-2__microtubule-associated protein 65-2	0.70
At5g43900	ATMYA2_MYA2_XI-2_XI-6__myosin 2	0.70
At4g14390	Ankyrin repeat family protein	0.75
At4g19660	ATNPR4_NPR4__NPR1-like protein 4	0.76
At3g24530	AAA-type ATPase family protein / ankyrin repeat family protein	0.77
At4g39320	microtubule-associated protein-related	0.81
At5g55400	Actin binding Calponin homology (CH) domain-containing protein	0.85
At2g31200	ADF6_ATADF6__actin depolymerizing factor 6	0.89
At3g13190	Plant protein of unknown function (DUF827)	0.91

TAIR "cell wall"

D 16h

Gene ID	Tair Annotation	Ratio
At5g65390	AGP7__arabinogalactan protein 7	-0.68
At3g11700	FLA18__FASCICLIN-like arabinogalactan protein 18 precursor	-1.06
At2g47930	AGP26_ATAGP26__arabinogalactan protein 26	-0.97
At2g23130	AGP17_ATAGP17__arabinogalactan protein 17	-0.79
At3g22440	FRIGIDA-like protein	-0.75
At2g28240	ATP-dependent helicase family protein	-0.87
At4g13340	LRX3__Leucine-rich repeat (LRR) family protein	-0.64
At5g49720	ATGH9A1_DEC_GH9A1_IRX2_KOR_KOR1_RSW2_TSD1__glycosyl hydrolase 9A1	-1.22
At5g05170	ATCESA3_ATH-B_CESA3_CEV1_IXR1__Cellulose synthase family protein	-1.62
At4g32410	AtCESA1_CESA1_RSW1__cellulose synthase 1	-1.10
At4g23990	ATCSLG3_CSLG3__cellulose synthase like G3	-1.16
At1g55850	ATCSLE1_CSLE1__cellulose synthase like E1	-0.71
At5g06860	ATPGIP1_PGIP1__polygalacturonase inhibiting protein 1	-0.83
At3g16850	Pectin lyase-like superfamily protein	-0.98
At1g19170	Pectin lyase-like superfamily protein	-0.69
At5g62620	Galactosyltransferase family protein	-1.68

At5g57550	XTH25_XTR3__xyloglucan endotransglucosylase/hydrolase 25	-0.71
At3g48580	XTH11__xyloglucan endotransglucosylase/hydrolase 11	-0.90
At3g61130	GAUT1_LGT1__galacturonosyltransferase 1	-0.72
At3g09410	Pectinacetylesterase family protein	-0.55
At2g46930	Pectinacetylesterase family protein	-0.75
At4g30440	GAE1__UDP-D-glucuronate 4-epimerase 1	-0.67
At3g23820	GAE6__UDP-D-glucuronate 4-epimerase 6	-0.66
At1g16340	ATKDSA2_ATKSDA__Aldolase superfamily protein	-0.57
At3g02570	MEE31_PMI1__Mannose-6-phosphate isomerase, type I	-0.56
At3g01640	ATGLCAK_GLCAK__glucuronokinase G	-0.61
At1g53500	ATMUM4_ATRHM2_MUM4_RHM2__NAD-dependent epimerase/dehydratase family protein	-0.54
At3g62830	ATUXS2_AUD1_UXS2__NAD(P)-binding Rossmann-fold superfamily protein	-1.60
At3g53520	ATUXS1_UXS1__UDP-glucuronic acid decarboxylase 1	-1.02

ND 16h

Gene ID	Tair Annotation	Ratio
At1g64670	BDG1_CED1__alpha/beta-Hydrolases superfamily protein	0.97
At3g58800	unknown protein; Has 75 Blast hits to 75 proteins in 23 species: Archae - 0; Bacteria - 0; Metazo	1.26
At5g03170	ATFLA11_FLA11__FASCICLIN-like arabinogalactan-protein 11	0.53
At5g10430	AGP4_ATAGP4__arabinogalactan protein 4	0.66
At2g14890	AGP9__arabinogalactan protein 9	0.68
At5g06390	FLA17__FASCICLIN-like arabinogalactan protein 17 precursor	0.78
At3g60900	FLA10__FASCICLIN-like arabinogalactan-protein 10	0.81
At3g13520	AGP12_ATAGP12__arabinogalactan protein 12	1.03
At5g60490	FLA12__FASCICLIN-like arabinogalactan-protein 12	1.09
At1g55330	AGP21_ATAGP21__arabinogalactan protein 21	1.11
At2g04780	FLA7__FASCICLIN-like arabinoogalactan 7	1.67
At4g12730	FLA2__FASCICLIN-like arabinogalactan 2	2.12
At4g31590	ATCSLC05_ATCSLC5_CSLC05_CSLC5__Cellulose-synthase-like C5	0.61
At5g22740	ATCSLA02_ATCSLA2_CSLA02_CSLA2__cellulose synthase-like A02	0.75
At3g28180	ATCSLC04_ATCSLC4_CSLC04_CSLC04_CSLC4__Cellulose-synthase-like C4	1.23
At5g09870	CESA5__cellulose synthase 5	0.61
At3g47000	Glycosyl hydrolase family protein	0.69
At3g47010	Glycosyl hydrolase family protein	0.79
At1g78060	Glycosyl hydrolase family protein	0.54
At5g10560	Glycosyl hydrolase family protein	0.55
At5g66460	AtMAN7_MAN7__Glycosyl hydrolase superfamily protein	0.58
At5g49360	ATBXL1_BXL1__beta-xylosidase 1	0.87
At1g02640	ATBXL2_BXL2__beta-xylosidase 2	1.80
At3g57790	Pectin lyase-like superfamily protein	0.57
At4g33440	Pectin lyase-like superfamily protein	0.60
At3g62110	Pectin lyase-like superfamily protein	0.62
At3g06770	Pectin lyase-like superfamily protein	0.63
At4g23820	Pectin lyase-like superfamily protein	0.70
At1g04680	Pectin lyase-like superfamily protein	0.89
At4g23500	Pectin lyase-like superfamily protein	0.91
At2g43860	Pectin lyase-like superfamily protein	0.96
At3g53190	Pectin lyase-like superfamily protein	1.08
At5g48900	Pectin lyase-like superfamily protein	1.08
At4g24780	Pectin lyase-like superfamily protein	1.29
At3g61490	Pectin lyase-like superfamily protein	1.41
At3g15720	Pectin lyase-like superfamily protein	1.87
At1g67750	Pectate lyase family protein	2.08
At5g22940	F8H__FRA8 homolog	1.23
At4g30290	ATXTH19_XTH19__xyloglucan endotransglucosylase/hydrolase 19	0.59
At4g28250	ATEXPB3_ATHEXP BETA 1.6_EXPB3__expansin B3	0.71
At4g03210	XTH9__xyloglucan endotransglucosylase/hydrolase 9	0.81
At1g11545	XTH8__xyloglucan endotransglucosylase/hydrolase 8	0.82
At2g06850	EXGT-A1_EXT_XTH4__xyloglucan endotransglucosylase/hydrolase 4	1.13
At5g02260	ATEXP9_ATEXPA9_ATHEXP ALPHA 1.10_EXP9_EXPA9__expansin A9	1.19
At5g05290	ATEXP2_ATEXPA2_ATHEXP ALPHA 1.12_EXP2_EXPA2__expansin A2	1.22
At2g37640	ATEXP3_ATEXPA3_ATHEXP ALPHA 1.9_EXP3__Barwin-like endoglucanases superfamily	1.22
At5g13870	EXGT-A4_XTH5__xyloglucan endotransglucosylase/hydrolase 5	1.32
At2g40610	ATEXP8_ATEXPA8_ATHEXP ALPHA 1.11_EXP8_EXPA8__expansin A8	1.39

At1g10550	XET_XTH33__xyloglucan:xyloglucosyl transferase 33	1.41
At1g26770	AT-EXP10_ATEXP10_ATEXPA10_ATHEXP ALPHA 1.1_EXP10_EXPA10__expansin A10	1.48
At4g30280	ATXTH18_XTH18__xyloglucan endotransglucosylase/hydrolase 18	1.58
At3g44990	AtXTH31_ATXTR8_XTH31_XTR8__xyloglucan endo-transglycosylase-related 8	1.67
At2g03090	ATEXP15_ATEXPA15_ATHEXP ALPHA 1.3_EXP15_EXPA15__expansin A15	1.68
At1g69530	AT-EXP1_ATEXP1_ATEXPA1_ATHEXP ALPHA 1.2_EXP1_EXPA1__expansin A1	1.71
At3g05910	Pectinacetylesterase family protein	0.91
At5g26670	Pectinacetylesterase family protein	0.94
At5g64640	Plant invertase/pectin methylesterase inhibitor superfamily	0.61
At3g49220	Plant invertase/pectin methylesterase inhibitor superfamily	0.75
At3g14310	ATPME3_PME3__pectin methylesterase 3	0.76
At3g10720	Plant invertase/pectin methylesterase inhibitor superfamily	0.88
At4g03926		1.31
At4g01750	RGXT2__rhamnogalacturonan xylosyltransferase 2	0.56
At1g73250	ATFX_GER1__GDP-4-keto-6-deoxymannose-3,5-epimerase-4-reductase 1	1.03
At3g54690	SETH3__Sugar isomerase (SIS) family protein	0.59
At1g79500	AtkdsA1__Aldolase-type TIM barrel family protein	0.69
At1g01220	AtFKGP_FKGP__L-fucokinase/GDP-L-fucose pyrophosphorylase	0.68
At5g39320	UDP-glucose 6-dehydrogenase family protein	1.12
At1g26570	ATUGD1_UGD1__UDP-glucose dehydrogenase 1	1.22
At1g64440	REB1_RHD1_UGE4__NAD(P)-binding Rossmann-fold superfamily protein	0.53
At2g47650	UXS4__UDP-xylose synthase 4	0.80

D 24h

Gene ID	Tair Annotation	Ratio
At3g11700	FLA18__FASCICLIN-like arabinogalactan protein 18 precursor	-0.79
At2g28240	ATP-dependent helicase family protein	-0.53
At5g49720	ATGH9A1_DEC_GH9A1_IRX2_KOR_KOR1_RSW2_TSD1__glycosyl hydrolase 9A1	-1.23
At4g07960	ATCSLC12_CSLC12_CSLC12__Cellulose-synthase-like C12	-0.52
At5g05170	ATCESA3_ATH-B_CESA3_CEV1_IXR1__Cellulose synthase family protein	-2.01
At4g32410	AtCESA1_CESA1_RSW1__cellulose synthase 1	-1.43
At4g23990	ATCSLG3_CSLG3__cellulose synthase like G3	-0.89
At5g34940	AtGUS3_GUS3__glucuronidase 3	-0.66
At5g64570	ATBXL4_XYL4__beta-D-xylosidase 4	-0.98
At1g67830	ATFXG1_FXG1__alpha-fucosidase 1	-0.51
At1g19170	Pectin lyase-like superfamily protein	-0.61
At2g43880	Pectin lyase-like superfamily protein	-0.53
At5g62620	Galactosyltransferase family protein	-1.71
At5g16890	Exostosin family protein	-0.55
At3g23730	XTH16__xyloglucan endotransglucosylase/hydrolase 16	-0.78
At5g57560	TCH4_XTH22__Xyloglucan endotransglucosylase/hydrolase family protein	-0.61
At5g39310	ATEXP24_ATEXPA24_ATHEXP ALPHA 1.19_EXP24_EXPA24__expansin A24	-0.51
At2g46930	Pectinacetylesterase family protein	-0.91
At1g56100	Plant invertase/pectin methylesterase inhibitor superfamily protein	-1.15
At4g02320	Plant invertase/pectin methylesterase inhibitor superfamily	-0.60
At4g33220	ATPME44_PME44__pectin methylesterase 44	-0.72
At1g11580	ATPMEPCRA_PMEPCRA__methylesterase PCR A	-0.71
At3g25140	GAUT8_QUA1__Nucleotide-diphospho-sugar transferases superfamily protein	-1.77
At3g61130	GAUT1_LGT1__galacturonosyltransferase 1	-0.93
At4g30440	GAE1__UDP-D-glucuronate 4-epimerase 1	-1.05
At3g23820	GAE6__UDP-D-glucuronate 4-epimerase 6	-0.61
At1g53000	AtCKS_CKS_KDSB__Nucleotide-diphospho-sugar transferases superfamily protein	-0.69
At1g16340	ATKDSA2_ATKSDA__Aldolase superfamily protein	-1.58
At3g02570	MEE31_PMI1__Mannose-6-phosphate isomerase, type I	-0.57
At3g01640	ATGLCAK_GLCAK__glucuronokinase G	-0.94
At1g63000	NRS/ER_UER1__nucleotide-rhamnose synthase/epimerase-reductase	-1.45
At1g12780	ATUGE1_UGE1__UDP-D-glucose/UDP-D-galactose 4-epimerase 1	-1.64
At1g63180	UGE3__UDP-D-glucose/UDP-D-galactose 4-epimerase 3	-1.54
At3g62830	ATUXS2_AUD1_UXS2__NAD(P)-binding Rossmann-fold superfamily protein	-1.41
At3g53520	ATUXS1_UXS1__UDP-glucuronic acid decarboxylase 1	-1.23

ND 24h

Gene ID	Tair Annotation	Ratio
At3g57690	AGP23_ATAGP23__arabinogalactan protein 23	0.68
At5g22740	ATCSLA02_ATCSLA2_CSLA02_CSLA2__cellulose synthase-like A02	0.66
At3g28180	ATCSLC04_ATCSLC4_CSLC04_CSLC04_CSLC4__Cellulose-synthase-like C4	0.81
At3g47000	Glycosyl hydrolase family protein	0.58
At5g66460	AtMAN7_MAN7__Glycosyl hydrolase superfamily protein	0.98

At5g06860	ATPGIP1_PGIP1__polygalacturonase inhibiting protein 1	0.52
At4g33440	Pectin lyase-like superfamily protein	0.75
At3g62110	Pectin lyase-like superfamily protein	1.04
At2g43860	Pectin lyase-like superfamily protein	1.06
At3g15720	Pectin lyase-like superfamily protein	1.10
At4g36890	IRX14__Nucleotide-diphospho-sugar transferases superfamily protein	0.64
At2g18660	AtPNP-A_PNP-A__plant natriuretic peptide A	0.53
At1g32170	XTH30_XTR4__xyloglucan endotransglucosylase/hydrolase 30	0.55
At5g05290	ATEXP2_ATEXPA2_ATHEXP ALPHA 1.12_EXP2_EXPA2__expansin A2	0.57
At2g39700	ATEXP4_ATEXPA4_ATHEXP ALPHA 1.6_EXPA4__expansin A4	0.62
At2g03090	ATEXP15_ATEXPA15_ATHEXP ALPHA 1.3_EXP15_EXPA15__expansin A15	0.70
At1g26770	AT-EXP10_ATEXP10_ATEXPA10_ATHEXP ALPHA 1.1_EXP10_EXPA10__expansin A10	0.73
At1g69530	AT-EXP1_ATEXP1_ATEXPA1_ATHEXP ALPHA 1.2_EXP1_EXPA1__expansin A1	0.97
At5g33290	XGD1__xylogalacturonan deficient 1	0.76
At1g79500	AtkdsA1__Aldolase-type TIM barrel family protein	0.81
At2g34850	MEE25__NAD(P)-binding Rossmann-fold superfamily protein	0.60
At1g30620	HSR8_MUR4_UXE1__NAD(P)-binding Rossmann-fold superfamily protein	0.81
At3g10700	GalAK__galacturonic acid kinase	0.52
At3g01010	UDP-glucose/GDP-mannose dehydrogenase family protein	0.52
