# Supplementary material for: Microtubule self-organisation during seed germination in Arabidopsis
Source: BMC Biol. 2020 Apr 30;18:44. doi: 10.1186/s12915-020-00774-8 (PMC7191766; doi:10.1186/s12915-020-00774-8)
Supplement: Supplementary file 4 — Additional file 4: Figure S3. Immunodetection of α tubulin. A 5 μg aliquot of total protein was loaded on each lane and α-tubulin was detected with α-tubulin antibody in dormant and non-dormant Arabidopsis seeds. HOI, hours of imbibition at 25°C in the dark; DOS, days of stratification at 4°C in the dark. (PPTX 935 kb) [file 12915_2020_774_MOESM4_ESM.pptx]

## Slide 1
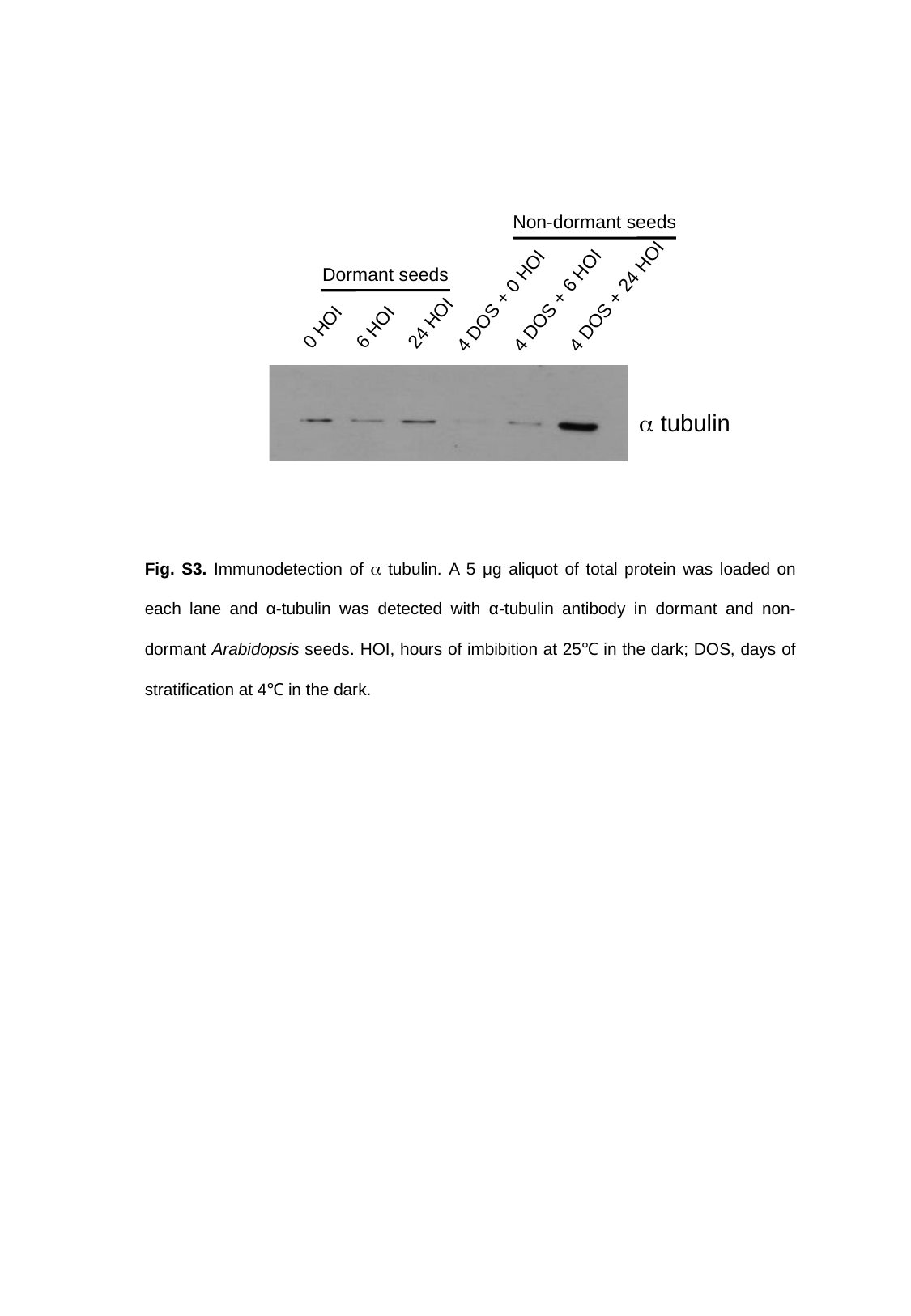

Non-dormant seeds
Dormant seeds
4 DOS + 24 HOI
4 DOS + 6 HOI
4 DOS + 0 HOI
24 HOI
0 HOI
6 HOI
α tubulin
Fig. S3. Immunodetection of α tubulin. A 5 μg aliquot of total protein was loaded on each lane and α-tubulin was detected with α-tubulin antibody in dormant and non-dormant Arabidopsis seeds. HOI, hours of imbibition at 25℃ in the dark; DOS, days of stratification at 4℃ in the dark.
