# Supplementary material for: Microtubule self-organisation during seed germination in Arabidopsis
Source: BMC Biol. 2020 Apr 30;18:44. doi: 10.1186/s12915-020-00774-8 (PMC7191766; doi:10.1186/s12915-020-00774-8)
Supplement: Supplementary file 5 — Additional file 5: Figure S4. Expression of tubulin and tubulin regulators genes in mutant seeds development. Expression of TUB3, TUB4, TUB5, TUB6, TUA6, MAPD65-1, MAP65-2, CLASP, KTN1, MOR1 and TBG1 in GA and ABA mutant seeds (indicated in figures). Data obtained through the Arabidopsis eFP browser at bar.toronto.ca. (PPTX 11238 kb) [file 12915_2020_774_MOESM5_ESM.pptx]

## Slide 1
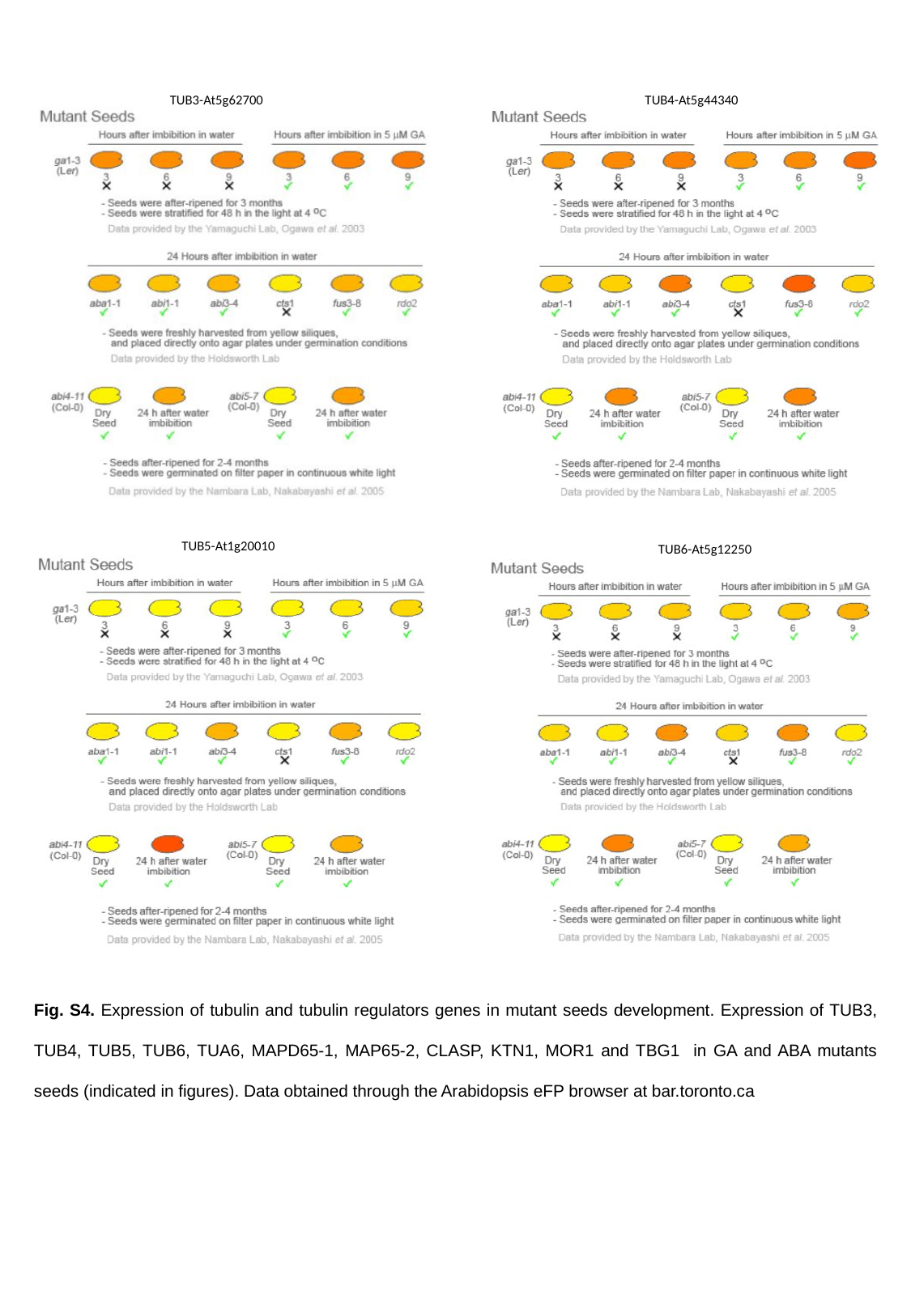

TUB3-At5g62700
TUB4-At5g44340
TUB5-At1g20010
TUB6-At5g12250
Fig. S4. Expression of tubulin and tubulin regulators genes in mutant seeds development. Expression of TUB3, TUB4, TUB5, TUB6, TUA6, MAPD65-1, MAP65-2, CLASP, KTN1, MOR1 and TBG1 in GA and ABA mutants seeds (indicated in figures). Data obtained through the Arabidopsis eFP browser at bar.toronto.ca

## Slide 2
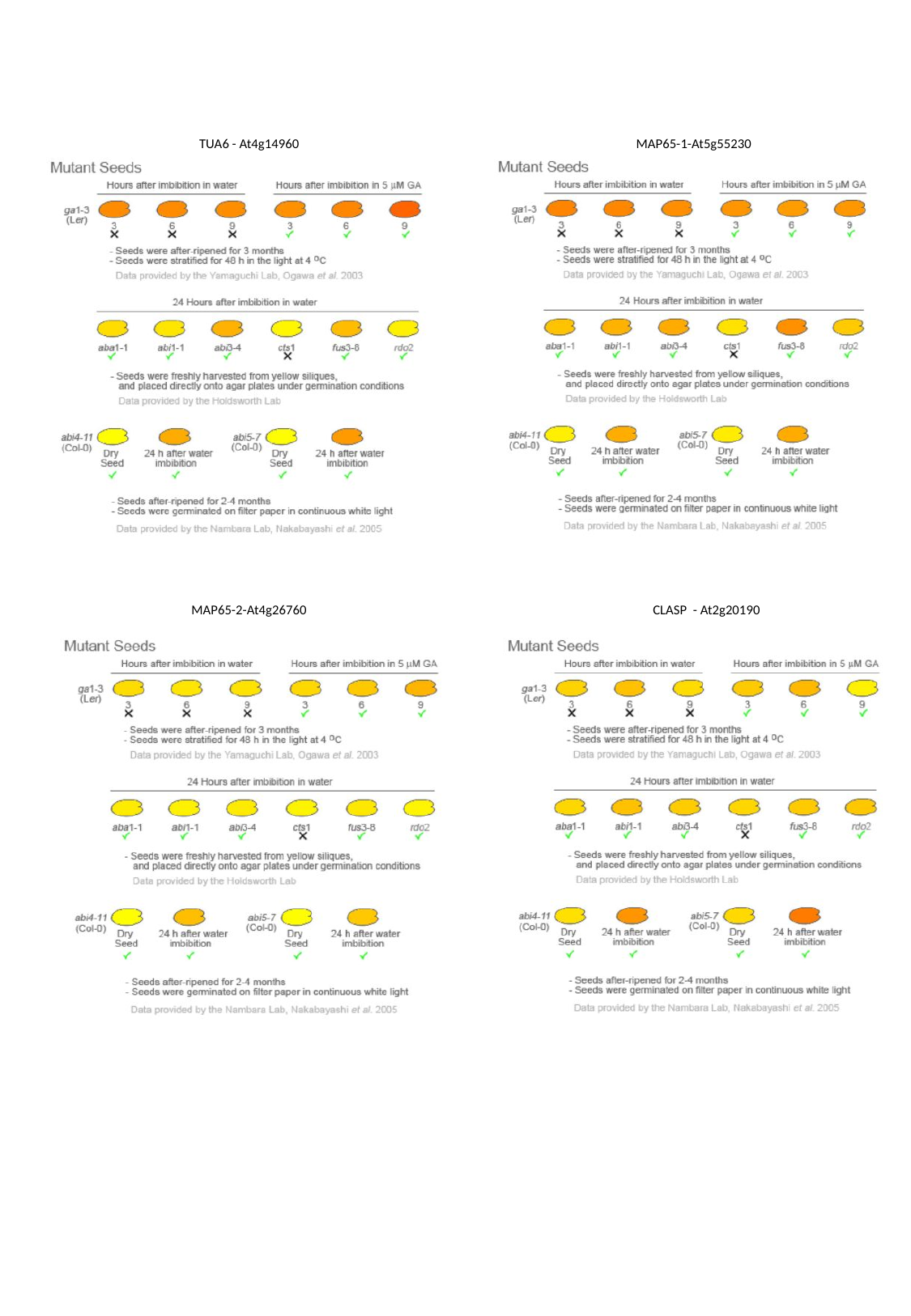

TUA6 - At4g14960
MAP65-1-At5g55230
MAP65-2-At4g26760
CLASP - At2g20190

## Slide 3
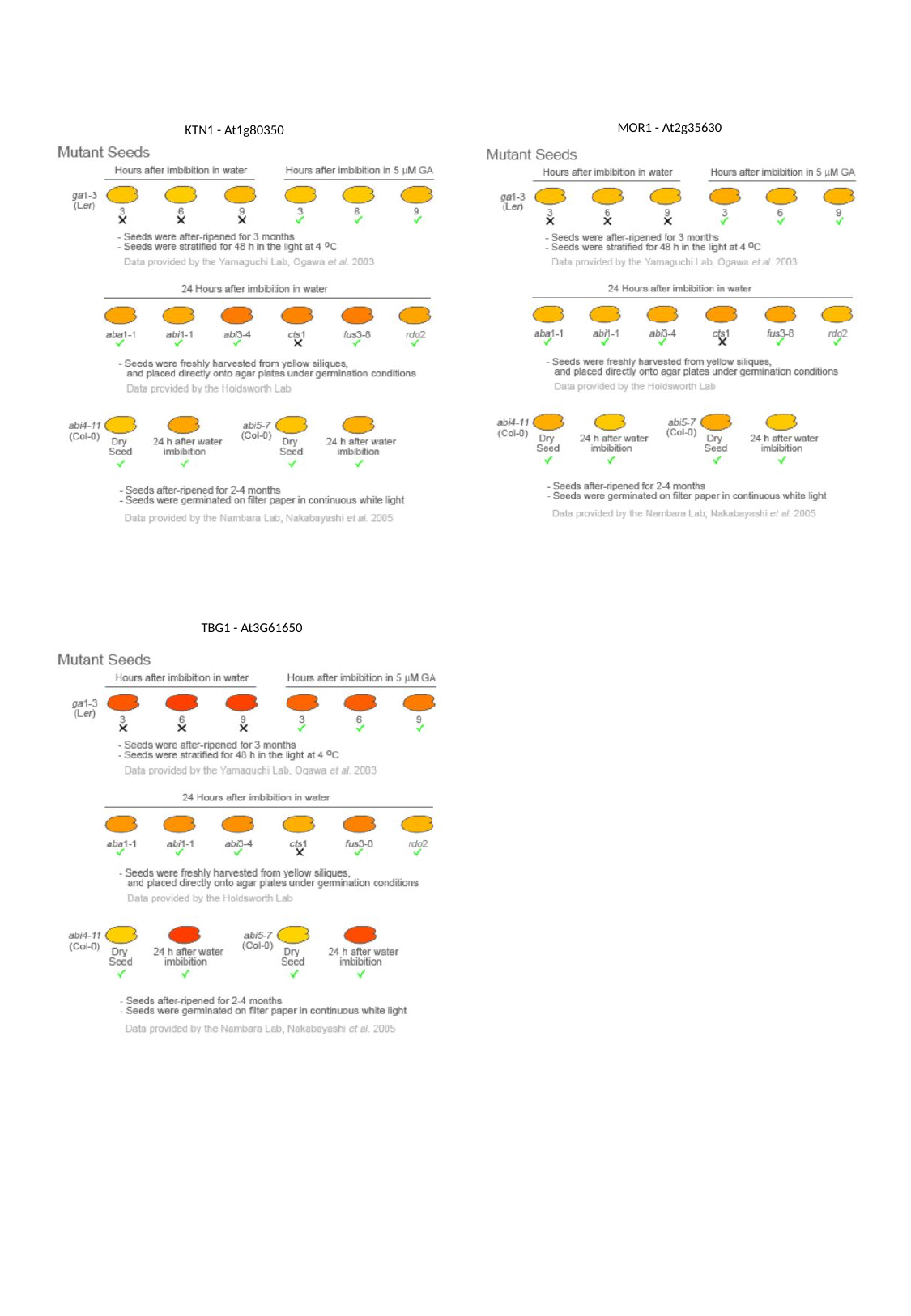

MOR1 - At2g35630
KTN1 - At1g80350
TBG1 - At3G61650
