# Supplementary material for: Microtubule self-organisation during seed germination in Arabidopsis
Source: BMC Biol. 2020 Apr 30;18:44. doi: 10.1186/s12915-020-00774-8 (PMC7191766; doi:10.1186/s12915-020-00774-8)
Supplement: Supplementary file 6 — Additional file 6: Table S2. Primer sequences used for qRT-PCR. [file 12915_2020_774_MOESM6_ESM.pdf]

Table S2. Primer sequences used for qRT-PCR.

Gene ID	Gene name	Forward primer (5'-3')	Reverse primer (5'-3')
At4g14960	*TUA6*	AACATGGCATTCAGCCTGAT	CGTCGATCACAGTTGGCTC
At5g62700	*TUB3*	CCCAGCTTTGGTGATTTGAAC	GCTTACGGAGGTCAGAGTTGA
At5g44340	*TUB4*	TCCTACCTTTGGTGATCTTAACCAT	CAACCATGAAGAAGTGAAGCCTTG
At1g20010	*TUB5*	CCTAGCTTTGGAGACTTGAACC	CCACCATGAAGAAATGGAGACGAG
At5g12250	*TUB6*	ACTCCTAGCTTTGGTGATCTGAAT	TGGAGACGAGGGAAAGGAATG
AT3G61650	*TUBG1*	AAGCTGATGGAAGTGACAGT	ATAAGATCCCATACCTGAGCCA
At5g55230	*MAP65-1*	CCAGAAAGAGAAGAGTGACAGG	GTGACGGTGCTTAAGAAATCCAA
At4g26760	*MAP65-2*	TCCAGAAAGAGAAGAGTGATAGGT	TCTAAGCTCGGATGAACTTCGG
At3g47690	*EB1a*	TCCGATAGTGGTAGCAGTGAAG	TTCCAGTTGTTCTCCGTTTCCT
At2g20190	*CLASP*	CTCTTGTTCCTGCTGTTGTCG	ATGAAACCTCCATGAGAGTCGT
At1g80350	*KTN1*	CTGAGTCTATGAATGGTGATGC	CATCCCAACGGACACCAG
AT2g35630	*MOR1*	GATTCTTGGTGCTGACATCTGG	CGCCTTAATGCTGCTCGT
At5g53560	*Cytochrome B5 isoform E*	TGAAGAAGTTTCAAAGCACAACA	TCATCCATGAATGGAGTCACA
At4g26410	*Chromosome-associated kinesin*	GAGCTGAAGTGGCTTCCATGAC	GGTCCGACATACCCATGATCC
At4g34270	*TIP41-like family protein*	GTGAAAACTGTTGGAGAGAAGCAA	TCAACTGGATACCCTTTCGCA
